# SAILoR: Structure-Aware Inference of Logic Rules

**DOI:** 10.1371/journal.pone.0304102

**Published:** 2024-06-11

**Authors:** Žiga Pušnik, Miha Mraz, Nikolaj Zimic, Miha Moškon

**Affiliations:** Faculty of Computer and Information Science, University of Ljubljana, Ljubljana, Slovenia; University of the Philippines Diliman, PHILIPPINES

## Abstract

Boolean networks provide an effective mechanism for describing interactions and dynamics of gene regulatory networks (GRNs). Deriving accurate Boolean descriptions of GRNs is a challenging task. The number of experiments is usually much smaller than the number of genes. In addition, binarization leads to a loss of information and inconsistencies arise in binarized time-series data. The inference of Boolean networks from binarized time-series data alone often leads to complex and overfitted models. To obtain relevant Boolean models of gene regulatory networks, inference methods could incorporate data from multiple sources and prior knowledge in terms of general network structure and/or exact interactions. We propose the Boolean network inference method SAILoR (Structure-Aware Inference of Logic Rules). SAILoR incorporates time-series gene expression data in combination with provided reference networks to infer accurate Boolean models. SAILoR automatically extracts topological properties from reference networks. These can describe a more general structure of the GRN or can be more precise and describe specific interactions. SAILoR infers a Boolean network by learning from both continuous and binarized time-series data. It navigates between two main objectives, topological similarity to reference networks and correspondence with gene expression data. By incorporating the NSGA-II multi-objective genetic algorithm, SAILoR relies on the wisdom of crowds. Our results indicate that SAILoR can infer accurate and biologically relevant Boolean descriptions of GRNs from both a static and a dynamic perspective. We show that SAILoR improves the static accuracy of the inferred network compared to the network inference method dynGENIE3. Furthermore, we compared the performance of SAILoR with other Boolean network inference approaches including Best-Fit, REVEAL, MIBNI, GABNI, ATEN, and LogBTF. We have shown that by incorporating prior knowledge about the overall network structure, SAILoR can improve the structural correctness of the inferred Boolean networks while maintaining dynamic accuracy. To demonstrate the applicability of SAILoR, we inferred context-specific Boolean subnetworks of female *Drosophila melanogaster* before and after mating.

## Introduction

Gene regulatory networks (GRNs) are part of a robust fundamental underlying system that controls the development, adaptability, and primary cellular processes in organisms [1]. The representation of GRNs with a set of binary variables and logical rules offers a simple and effective method to describe and characterize such systems and their dynamics with basic building blocks and modular substructures [2]. In a Boolean network, each gene is represented as a binary variable that is regulated by a set of regulators via logical rules. As a result, each gene can either be completely expressed, representing the ON state, or completely silenced, representing the OFF state. The description of GRNs with Boolean models offers several advantages. Simulating the dynamics of GRNs with a Boolean network does not require additional information, such as values of kinetic parameters, other than logical rules and an initial state. Moreover, by describing gene dynamics with logical rules we can interpret the dynamics of the system and straightforwardly uncover regulatory interactions. Kauffman demonstrated that randomly constructed Boolean networks, in which each gene is regulated by two or three Boolean variables form short stable cycles that are found in regulatory systems of biological organisms [3].

Many computational methods for inference of Boolean networks from time-series data have been proposed. These utilize either heuristic algorithms [4–6], entropy and mutual information [7, 8], or machine learning approaches [9, 10]. For example, Li et al. [10] developed LogBTF, which utilizes regularized logistic regression to obtain regression coefficients, which act as weights in the Boolean threshold network model. The utilization of Boolean threshold functions for the description of GRNs is supported by recent analyses [11, 12] of expert-curated Boolean network models, which revealed a high prevalence of nested canalizing functions, where specific values of single inputs with different priorities determine the function value. Other tools have been developed for detailed investigation of Boolean network models. For example, Weidner et al. [13] developed the GatekeepR application, which identifies nodes in a Boolean network, whose perturbations will likely have a large effect on the system’s dynamics.

The majority of Boolean inference approaches infer logical rules from binarized time-series data. Inconsistencies in the training dataset can occur due to the loss of information in the process of binarization [14]. Consequently, the main underlying problem almost all Boolean inference approaches tackle is solving inconsistency problems, where the goal is to find a Boolean network with minimal dynamical error for the binarized time-series data (e.g. see [15]). While in general, these approaches perform decently in terms of inferring systems’ dynamics with logical rules, their ability to uncover accurate underlying influence graphs, i.e. static networks, is limited [16].

A recent review and benchmarking of approaches for Boolean inference of gene regulatory networks indicated that Boolean inference approaches tend to perform well in terms of dynamic accuracy and poorly in terms of static accuracy, i.e. structural correctness [16]. To alleviate these problems we suggested learning from continuous and binarized data in addition to incorporating prior knowledge in terms of the known general structure of gene regulatory networks.

The inference of biologically viable and accurate GRN models is still a challenging task. In addition to methods for the inference of Boolean networks, several GRN inference approaches have been proposed, including fuzzy logic-based approaches [17, 18], regression and machine learning-based approaches [19, 20], information theory-based approaches [21, 22], correlation-based approaches [23], and probabilistic approaches [24, 25]. Gene expression is a product guided by multiple processes and factors. The balance of transcripts and proteins is achieved with a complex transcriptional, post-transcriptional, translational, and post-translational regulation [26]. Therefore, the inference of regulatory relationships from transcriptomic data alone offers a rudimentary view of a system. Recently an effort has been made to incorporate multiple data sources while inferring regulatory interactions [27]. For example, somatic mutations [24] and other multi-omics data, e.g. copy number variations and DNA methylations [28], were integrated into the process of inferring gene regulatory networks. Seçilmiş et al. showed that the knowledge of the perturbation design matrix increases network inference accuracy [29, 30].

In the context of inferring Boolean rules, prior knowledge has already been applied, either in terms of partial network structure, dynamical constraints, or prior logic rules [31–33]. These methods, however, do not incorporate prior knowledge about a general network structure, e.g. motifs, network densities, and shallow network structure, GRNs ubiquitously have in common [34].

**Fig 1:**
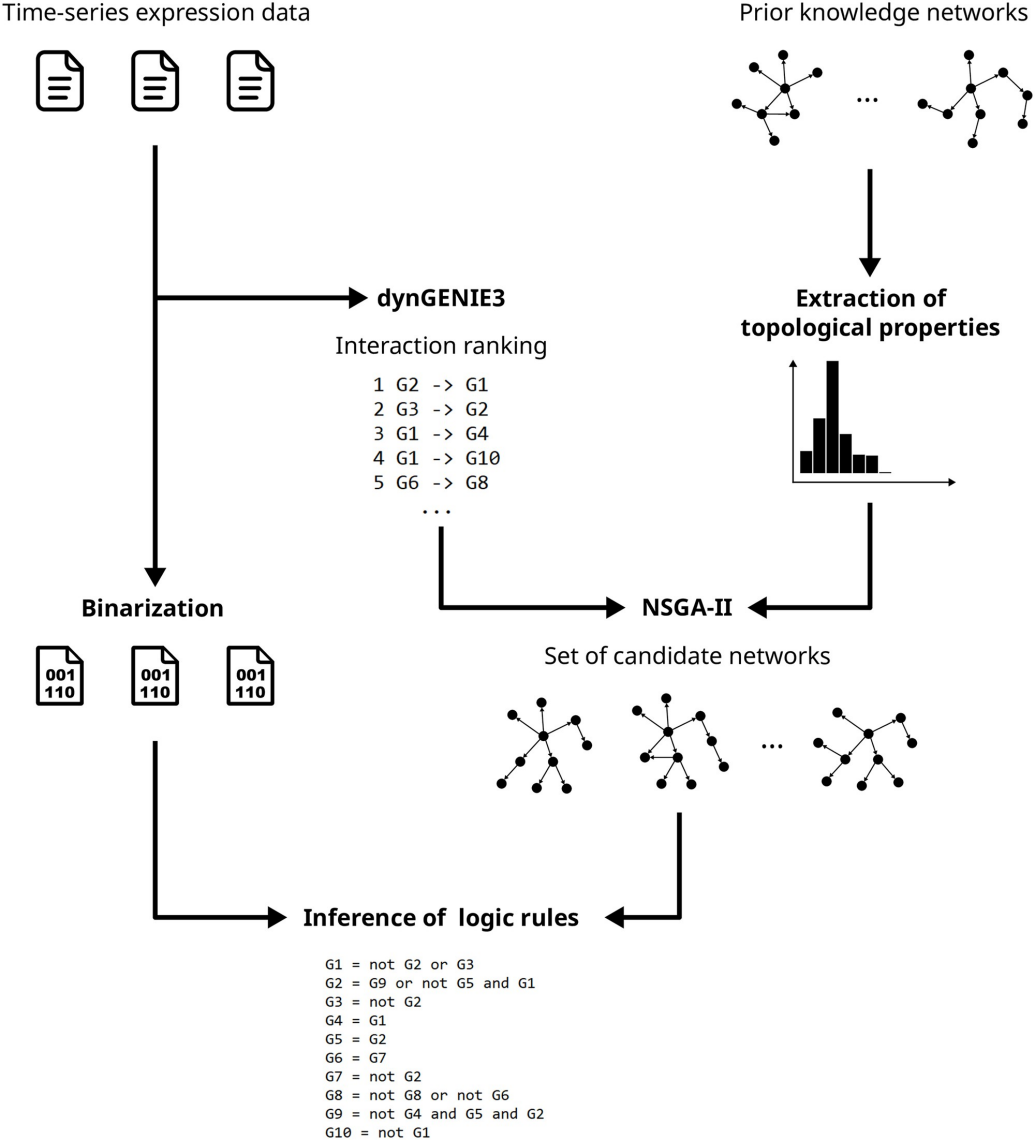
SAILoR flowchart. The figure represents a sequence of steps conducted in SAILoR.

Here, we introduce SAILoR (Structure-Aware Inference of Logic Rules), an inference method of gene regulatory systems described by Boolean networks. SAILoR utilizes prior knowledge in terms of provided reference networks from which topological properties are extracted. In contrast, to the majority of Boolean inference approaches that infer logic rules from binarized data, SAILoR infers logical rules from binarized as well as from continuous time-series data and by incorporating prior knowledge in terms of general network structure. SAILoR is structure-aware in terms that it generates a set of directed networks, which represent candidates for the influence graph of the final Boolean network. By utilizing the genetic algorithm NSGA-II [35], SAILoR generates network candidates that maximize the similarity to expected networks’ properties and compliance with the initial regulatory ranking, which are obtained with the inference method dynGENIE3 [20]. SAILoR automatically extracts expected network density, in-degree distribution, out-degree distribution, and triadic census (count of all connected triplets) from reference networks (see Fig 1).

Integration of prior knowledge in the form of context-aware reference networks and context-specific gene expression time-series data, allows SAILoR to infer accurate context-specific Boolean descriptions of GRNs. With context-aware Boolean GRN models, we can better predict the response of biological systems under different circumstances. Certain frameworks for sex-specific or disease-specific inference of static networks and Boolean models have already been proposed [33, 36, 37]. However, SAILoR is unique in the regard that the topological properties of reference networks combined with binarized time-series data directly influence the inference of Boolean models.

We assess the performance of SAILoR in different scenarios. First, we assess the ability of SAILoR to apply and utilize prior knowledge information to infer accurate static networks. We evaluate SAILoR on networks extracted from the *E. coli* gene regulatory network with GeneNetWeaver [38]. We then compare the predicted static network with the method dynGENIE3. Our results indicate a significant improvement in static accuracy for most cases (see section Structural properties of inferred networks). Further, we evaluate the ability of SAILoR to infer accurate Boolean networks. We compare the performance of SAILoR with other Boolean network inference approaches some of which were recently assessed in [16]. Our results indicate that the incorporation of prior knowledge in terms of general network structure allows SAILoR to improve the structural correctness of inferred Boolean networks while maintaining dynamic accuracy (see section Dynamic properties of inferred networks). Finally, we apply SAILoR to infer the context-specific Boolean subnetworks of *Drosophila melanogaster* before and after mating (see [39]).

In Background, we provide additional motivation for the utilization of prior knowledge information in terms of expected topological properties (section Topologies of gene regulatory networks), and provide a background for the genetic algorithm with non-dominated sorting (section Non-dominated sorting genetic algorithm NSGA-II). In section Materials and Methods, we describe SAILoR in detail, its crossover and mutation operators (section Crossover and mutation), and its optimization objectives (section Optimization objectives). In section Triadic census and dynamic update procedure we explain the algorithm for computing the triadic census and its dynamic update scheme, which significantly reduces time complexity. The procedure for the selection of the final Boolean network and the inference of Boolean rules is explained in section Selection of the optimal Boolean network. In addition, we describe our dataset and a benchmarking procedure in section Hyperparameter tuning and assessment of SAILoR. In section Structural properties of inferred networks, we demonstrate the applicability of SAILoR for the inference of static networks. The ability of SAILoR to infer accurate Boolean networks is demonstrated in section Dynamic properties of inferred networks. Furthermore, we analyze the computational complexity of SAILoR in section Computational complexity. In section Inference of context-specific gene regulatory subnetworks of *Drosophila melanogaster* we utilize SAILoR to infer the context-specific Boolean networks of *D. melanogaster*. We discuss the results, limitations, and advantages of SAILoR in section Conclusions.

## Background

### Topologies of gene regulatory networks

Gene regulatory networks are composed of frequently occurring regulation patterns named motifs, that guide the overall structure of gene regulatory networks [40]. Motifs in gene regulatory networks occur significantly more frequently than expected in randomly constructed networks. Two examples of frequently occurring motifs in gene regulation are feed-forward loop and bi-fan [34, 40, 41]. The same patterns occur in different kingdoms of taxonomic rank due to their universal capability to process information and optimally perform various functions [40]. For example, the feed-forward loop can act as a sign-sensitive delay [42], while regulated genes in bi-fan have a shared global function.

Nonetheless, the representation and occurrence of network motifs, do not include less frequent and average network patterns and can be thus ineffective for network analysis and feature extraction tasks [43]. Instead, small connected non-isomorphic induced subgraphs are often considered. For example, Pržulj [43] introduced network similarity metric based on graphlet degree distribution, by measuring the number of nodes touching each of the 73 possible orbits for 2, 3, 4, and 5 node graphlets. Pržulj showed, that many protein-protein interaction networks have high similarity with geometric random graphs. Metrics based on graphlets capture additional network properties that motifs might miss. More often graphlets are utilized in network embedding algorithms to learn feature representation (e.g., see gl2vec [44]) or to produce graphlet-based graph kernels [45, 46].

In addition, gene regulatory networks have similar general properties including a shallow structure, small average path length, and in-degree and out-degree distributions [47]. Large and complex networks occurring in nature are often termed scale-free networks due to the scale-free property of node degree distributions. In scale-free networks node degree distribution follows a power law, where the fraction of nodes with *k* edges decreases by *p*(*k*) ≈ *k*^−*γ*^ [41], where *γ* is some constant. This means, that a small fraction of nodes will act as hubs with many connections, while the majority of nodes will have few connections. Gene regulatory networks tend to have scale-free out-degree distribution, while the in-degree distribution is more restricted. This indicates that the regulation of multiple targets is more common than the regulation by many transcription factors [47].

### Non-dominated sorting genetic algorithm NSGA-II

Metaheuristic-based approaches are commonly used to find suboptimal solutions to difficult problems in a reasonable time [48]. Metaheuristics based on evolutionary strategies, such as genetic algorithms, are often considered. Many real-world problems require the identification of suboptimal points, where the tradeoff between multiple objectives is explored [49]. For these subproblems, multi-objective genetic algorithms based on non-dominated sorting (NSGA) have been shown to be very effective given a reasonable number of objectives [35, 50].

The use of multiple optimization objectives often leads to conflicting scenarios where the improvement of one objective leads to a reduction of other objectives. For this reason, the set of nondominated solutions is considered, where optimal solutions lie on a hypersurface known as the first Pareto frontier [51]. One solution dominates the other if it is better in at least one of the objectives and/or equal in others. Otherwise, two solutions are said to be non-dominated. Non-dominated sorting genetic algorithms are based on sorting solutions into separated non-dominated sets of solutions. In each iteration, only the best non-dominated solutions are selected. When a single non-dominated set is split to keep the predefined population size, the diversity measure is considered to maintain a diverse population. Srinivas and Deb [50] proposed NSGA with a diversity measure based on the shared fitness function, where the corresponding fitness value is reduced for subjects that lie in densely populated areas and vice versa. Deb et al. later in NSGA-II [35] introduced the diversity measure crowding-distance based on the cuboid defined by the subject’s neighboring solutions. In addition, Deb et al. improved the computational complexity of NSGA-II with fast non-dominated sorting. By increasing the number of objectives non-dominated sorting algorithms face numerous challenges. The evaluation of multiple objectives and the diversity measure can become computationally expensive, visualization of the Pareto frontier in higher dimensions is difficult and impractical, and the fraction of non-dominated solutions in the first Pareto frontier increases with the number of objectives, rendering sorting based on non-dominated levels inefficient [52]. NSGA-III [52] alleviates these problems to some extent by performing selection in the last Pareto frontier based on well-spread reference points, which can either be provided by the user or automatically generated and then updated in each generation. Automatically generated reference points are placed on a normalized hyperplane. The selection depends on the number of solutions associated with each reference point.

## Materials and methods

SAILoR automatically extracts expected in-degree and out-degree distributions, the expected count of connected triplets, i.e. triadic census, and the expected network density from reference networks. The method utilizes prior knowledge to generate a set of candidate networks, from which the best Boolean network is inferred. In a sense, SAILoR first performs an extensive feature selection procedure to infer potential gene regulators. Several Boolean methods already perform feature selection before the inference of logic rules (e.g. see [8, 9, 53]). However, instead of learning from binarized data, SAILoR performs inference of Boolean networks from continuous time-series data by applying the inference method dynGENIE3 [20], which produces a ranking of potential regulators. dynGENIE3 produces a matrix composed of regulatory weights from time-series data by regarding each time point as a separate steady-state condition. Regulation of each gene is modeled with a set of separate differential equations and the whole dynamics are estimated with a Random forest model. Finally, the weight of regulation of each gene is estimated from variable importance scores. Since regulatory weights obtained with dynGENIE3 do not have any statistical meaning, SAILoR operates with regulatory ranks. Given this ranking and/or expected in-degree distribution, the initial set of directed networks is constructed. SAILoR constructs the initial set of candidate networks by either selecting top regulations given the expected network density or by selecting top *k* regulators of each gene following the in-degree distribution, where the maximal value of *k* is limited to 10. When we have high confidence in the provided reference networks, SAILoR can generate initial subjects directly from such networks. However, in this case, the number of genes and their names must exactly match. The generated set of candidate networks represents the initial population of subjects in the genetic algorithm, which explores a set of Pareto optimal candidate networks. Through the use of NSGA-II SAILoR optimizes two main objectives, the topological similarity of candidate networks to reference networks and the compliance with the produced ranking based on the provided time-series data.

In the final step, SAILoR infers Boolean networks from optimal Pareto solutions, binarizing time-series gene expression data and generating generalized truth tables from which final logic rules are inferred with the Quine–McCluskey minimization method. While the Quine–McCluskey method has exponential computational complexity concerning the number of regulators, SAILoR limits the number of regulators. This constraint can be justified by the scale-free property of biological networks. The out-degree distribution of gene regulatory networks follows scale-free distribution, while the in-degree distribution follows a more restricted exponential function [47]. Recent reviews and analyses of biological Boolean network models have shown that only a small fraction of logic functions consists of 10 or more regulators (see [11, 12]). For example, Mitra et al. analyzed three datasets of curated Boolean models in which only 60 out of 8, 871 Boolean functions have more than 10 inputs [12]. Furthermore, in our case, the means of in-degree distributions of inferred Boolean functions are far below the predefined maximum number of regulators (see section Dynamic properties of inferred networks). The Boolean network that best matches binarized time-series data is selected as final. In the case, where multiple candidate networks have the same dynamic accuracy, one with the smallest Euclidean distance to the ideal solution in the normalized objective space is preferred. The data flow of SAILoR depicting primary steps is displayed in Fig 1. SAILoR is implemented in Python using the evolutionary computation framework DEAP [54]. SAILoR is available under the open-source MIT license on a GitHub repository https://github.com/zigapusnik/SAILoR and as a Docker image available on Docker Hub https://hub.docker.com/r/zigapusnik/sailor.

### Crossover and mutation

NSGA-II describes the selection operator defined by elitism, crowding distance, and multiple objectives. The user can define other additional operators. To generate a diverse population, SAILoR uses custom crossover and mutation.

**Fig 2:**
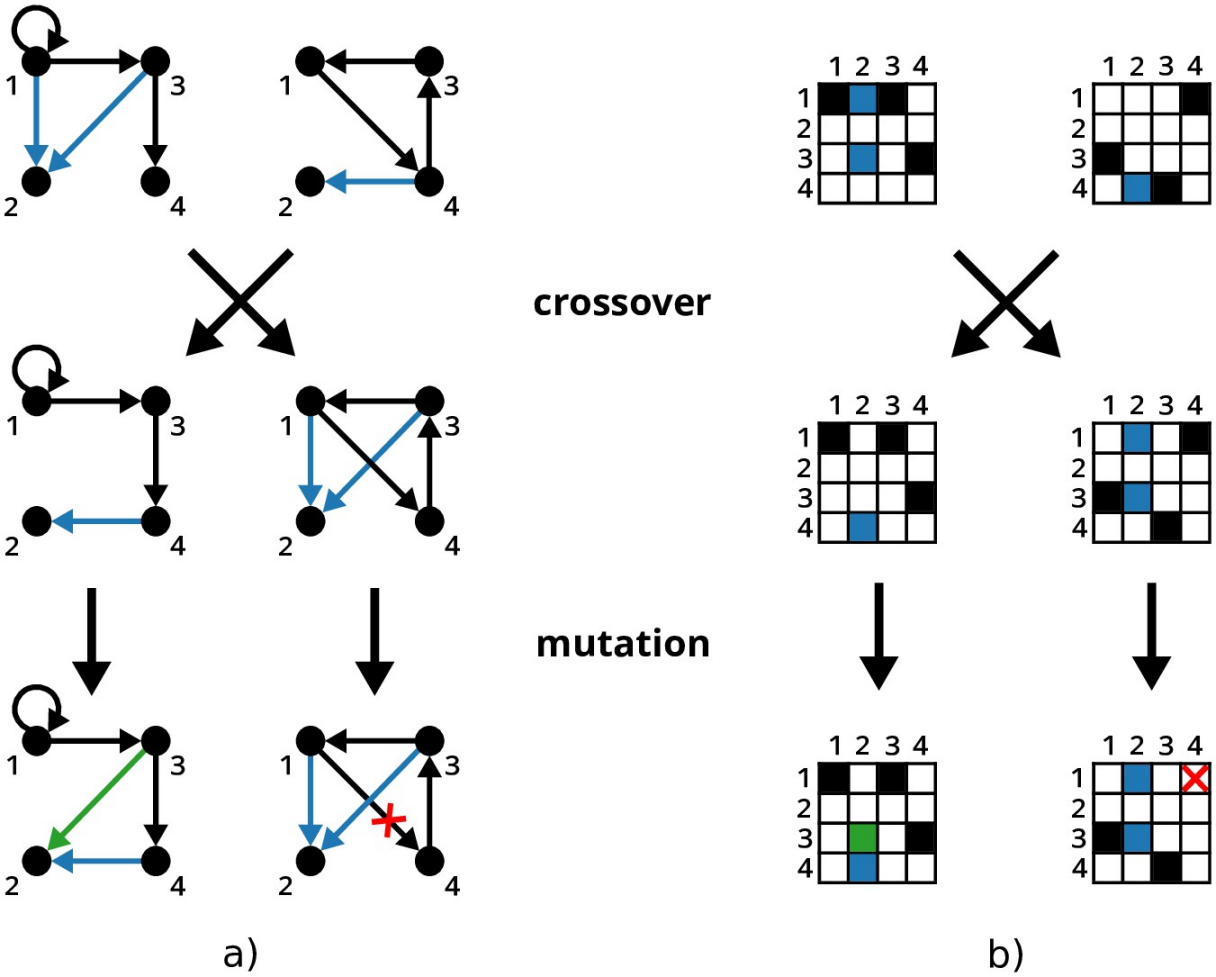
Depiction of crossover and mutation operators for two four node networks. Two distinct child networks are produced by exchanging regulators of the second node. In the next step, each offspring network is mutated. Fig a) illustrates the topology of given networks. Fig b) illustrates corresponding adjacency matrices. Blue edges are modified by crossover. Edges marked with green and red are modified by mutation.

We have implemented the derivation of *n*-point crossover, where two new subjects are generated by exchanging regulators for a random number of genes, which is achieved by exchanging columns of the adjacency matrices of the subjects (see Fig 2). In contrast to the exchange of regulation targets, i.e. rows in adjacency matrices, crossover with the exchange of regulators can gradually produce hub genes with high out-degree.

While the use of crossover can lead to large jumps in search space, the use of mutation typically promotes fine-grained exploration in local areas. In SAILoR, each individual is mutated by either adding or removing a single random edge in a network (see Fig 2). The probability of adding or removing an edge is the same. If the number of regulators after mutation exceeds the predefined maximum number, the effect of mutation is reversed. An advantage of mutating a single edge is that all topological properties can be upon mutation updated dynamically rather than calculated from scratch (see section Triadic census and dynamic update procedure).

In addition, mutation by adding or removing a single edge preserves the network densities, while mutation of multiple edges may not. For example, consider the mutation operator where the probability of flipping each element is *p*. The sequential use of this operation will produce subjects with approximate network densities of 1/2, regardless of the number of edges in initial networks and the value of *p*. Consider the adjacency matrix of a network with *n* × *n* elements and *a*_0_ ones. The number of ones in *k*-th iteration of mutation is then defined by (Eq 1). Let the term 1 − 2*p* be *q* and *pn*^2^ be *d*. Considering that |*q*| < 1 the limit when *k* goes to infinity is then equal 1/2 (see Eq 2).

$$\begin{array}{c} a_{k}=a_{k-1}\left(1-2p\right)+pn^{2} \end{array}$$ (1)

$$\begin{array}{c} \underset{k\to \infty }{\text{lim}}\;a_{0}q^{k}+d\left(q^{k-1}+q^{k-2}+\ldots +1\right)=\frac{d}{1-q}=\frac{n^{2}}{2} \end{array}$$ (2)

A simplified example portraying crossover and mutation operators employed by SAILoR is depicted in Fig 2. Offspring networks are produced by swapping regulators for random target genes. In the second step, each child network is additionally mutated.

### Optimization objectives

SAILoR applies the rank-based loss function (Eq 3) to measure how well a candidate network aligns with the regulatory ranks obtained with the base inference method dynGENIE3

$$\begin{array}{c} z_{1}\left(\hat{x}\right)=L\left(\hat{x},x\right)=\frac{2\sum_{c\in E}\text{rank}\left(e\right)}{\left|E\right|+{\left|E\right|}^{2}}-\frac{2\sum_{m\in E^{C}}\text{rank}\left(m\right)}{|E^{C}\left|+\right|E^{C}{\left||V\right|}^{2}+{\left|E^{C}\right|}^{2}}, \end{array}$$ (3)

where *E* is a set of all connections, i.e. regulations and *E*^*C*^ is a set of all missing connections, i.e. non-regulations. The set *V* contains the vertices of the network. The above equation is divided into two parts. The first part minimizes the sum of the ranks of regulations (rank(*e*)), while the second part maximizes the sum of the ranks of non-regulations (rank(*m*)). Both terms are divided by the sum of the top best ranks for regulations and the sum of the worst ranks for non-regulations.

SAILoR evaluates the topological similarity between reference and candidate networks based on multiple topological properties. Properties described with frequency distribution are assessed based on the histogram similarity with an overlap-based loss function. Utilization of overlap-based loss function is inspired by the Sørensen–Dice coefficient defined as $2|\hat{x}\cap x|/(|\hat{x}\left|+|x|\right)$ and Jaccard index similarly described as $|\hat{x}\cap x|/|\hat{x}\cup x|$ [55]. Both metrics are frequently used in machine vision to evaluate segmentation performance [55].

**Fig 3:**
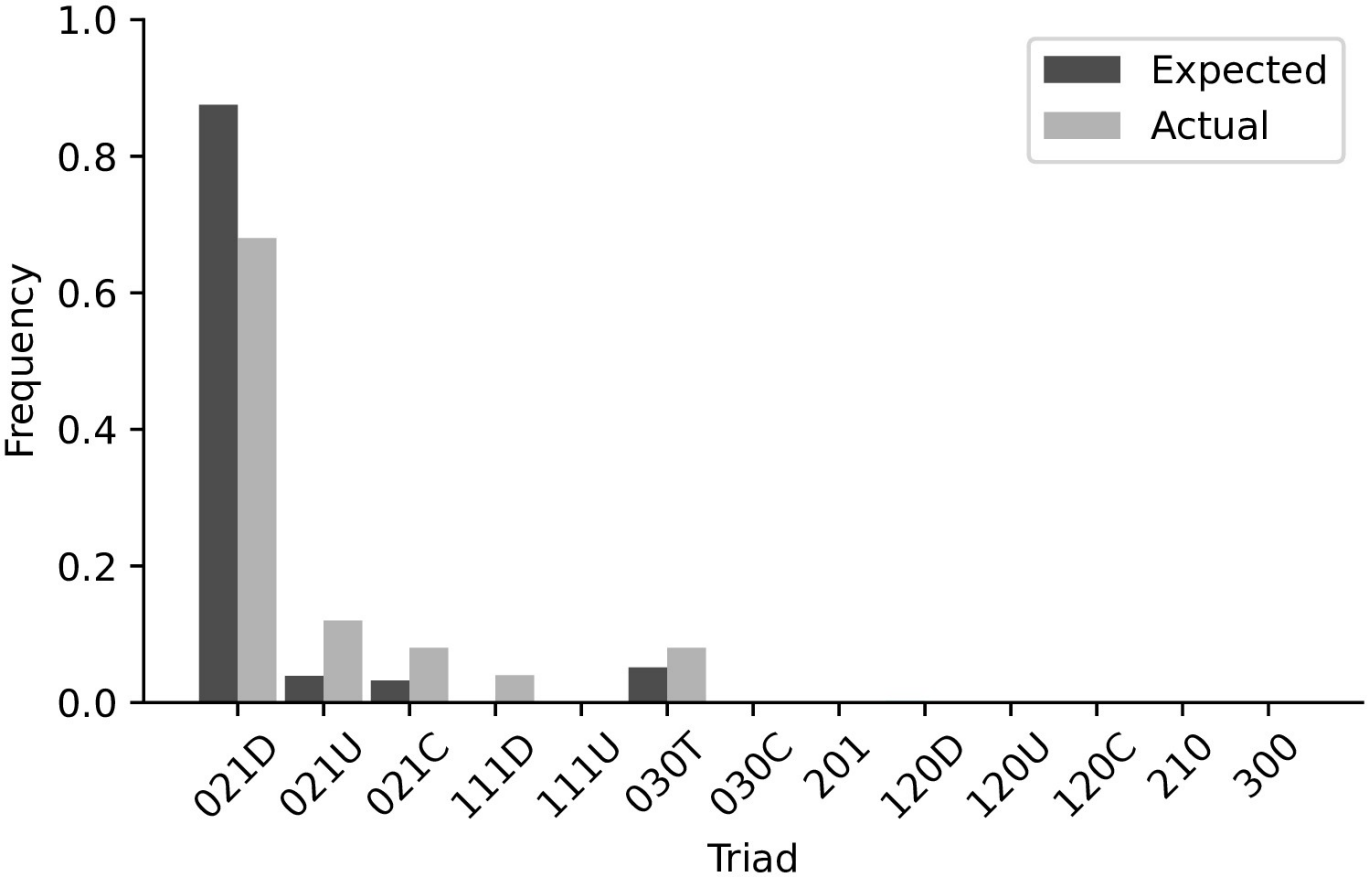
Example of expected and actual normalized triad frequencies. Example of possible triad frequencies obtained from the tenth generation of NSGA-II for a network with 16 nodes.

We observed the sparsity of inferred networks, in-degree distribution, out-degree distribution, and induced subgraphs with three connected nodes, i.e. triadic census with connected triads (see section Triadic census and dynamic update procedure). All topological properties are dynamically updated after mutation, rather than recalculated every time. Loss functions derived from the observed ($\hat{x}$) and expected distribution (*x*) are defined as an overlap between the histograms of the two

$$\begin{array}{c} L_{1,2,3}\left(\hat{x},x\right)=1-\sum_{i}min\left({\hat{x}}_{i},x_{i}\right), \end{array}$$ (4)

where *L*_1_ represents the loss based on the out-degree distribution, *L*_2_ is the loss function based on the in-degree distribution and *L*_3_ represents the loss function of the normalized triadic census. The in-degree is constrained by a predefined value of 10 regulators in different steps of our implementation. The out-degree in SAILoR is not limited, while it is evaluated for at most 10 connections. All nodes with a higher out-degree are thus considered to be nodes with an out-degree 10. We normalized the histogram of the triadic census with the sum of all triad counts. An example of expected and actual normalized triad frequencies is displayed in Fig 3.

**Fig 4:**
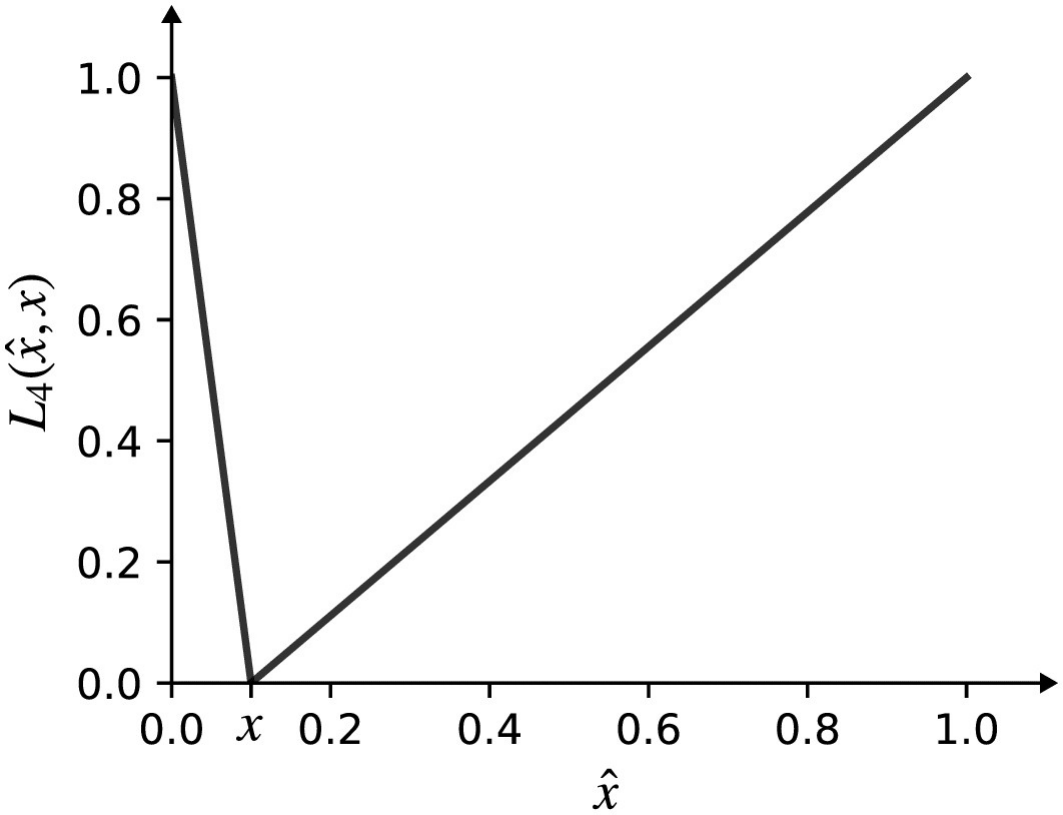
V-shaped piecewise linear function. Visualization of the loss function that describes the difference between the observed ($\hat{x}$) and the expected (*x*) network density.

The loss function describing the difference between the observed ($\hat{x}$) and expected edge probability (*x*) is a V-shaped piecewise function with different slopes (see Fig 4).

$$\begin{array}{c} L_{4}\left(\hat{x},x\right)=\{\begin{array}{cc} |x-\hat{x}|/x\text{,}\;\;\; & 0<\hat{x}<x\\ |x-\hat{x}|/\left(1-x\right)\text{,}\; & x\leq \hat{x}<1. \end{array} \end{array}$$ (5)

**Fig 5:**
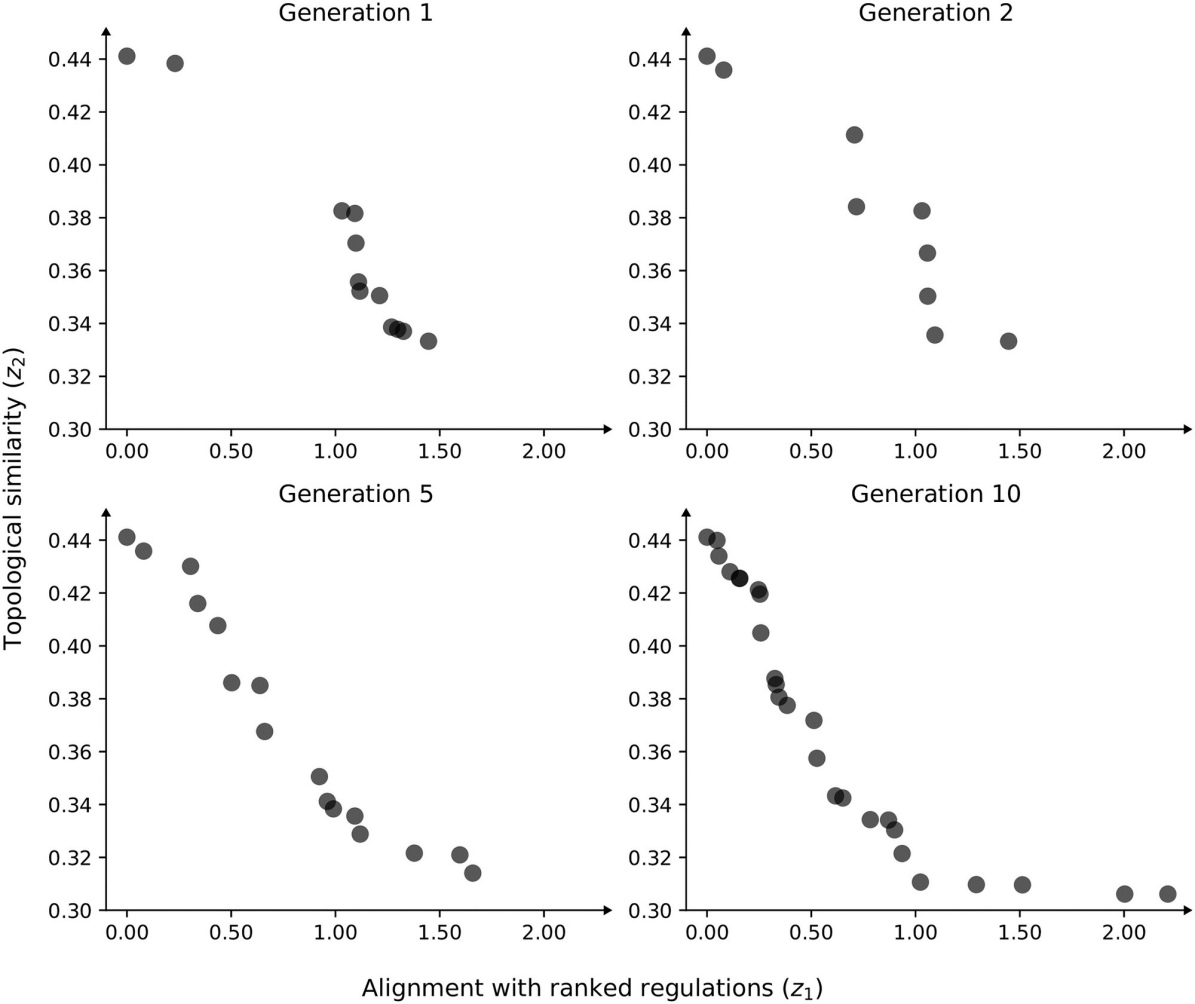
Evolution of Pareto frontier. Example of the improvement of the Pareto frontier through 10 NSGA-II generations on the network with 64 nodes. Each point represents a separate candidate network described by its adjacency matrix.

A positive aspect of the above function when inferring logical rules is that it slightly favors denser networks than expected. Finally, the total topological cost is evaluated as the weighted sum of partial topological loss functions (Eq 6) serving as a separate objective in the process of multiobjective optimization. The evolution of solutions in the first Pareto frontier with respect to optimization objectives is displayed in Fig 5.

$$\begin{array}{c} z_{2}\left(\hat{x}\right)=\alpha_{1}L_{1}\left(\hat{x},x\right)+\alpha_{2}L_{2}\left(\hat{x},x\right)+\alpha_{3}L_{3}\left(\hat{x},x\right)+\alpha_{4}L_{4}\left(\hat{x},x\right) \end{array}$$ (6)

Values of all partial topology-based loss functions range from 0 to 1. In a similar fashion we limited *α*_*i*_ weights from (Eq 6) to the interval [0, 1]. We performed a randomized search to obtain the optimal *α* values.

### Triadic census and dynamic update procedure

**Fig 6:**
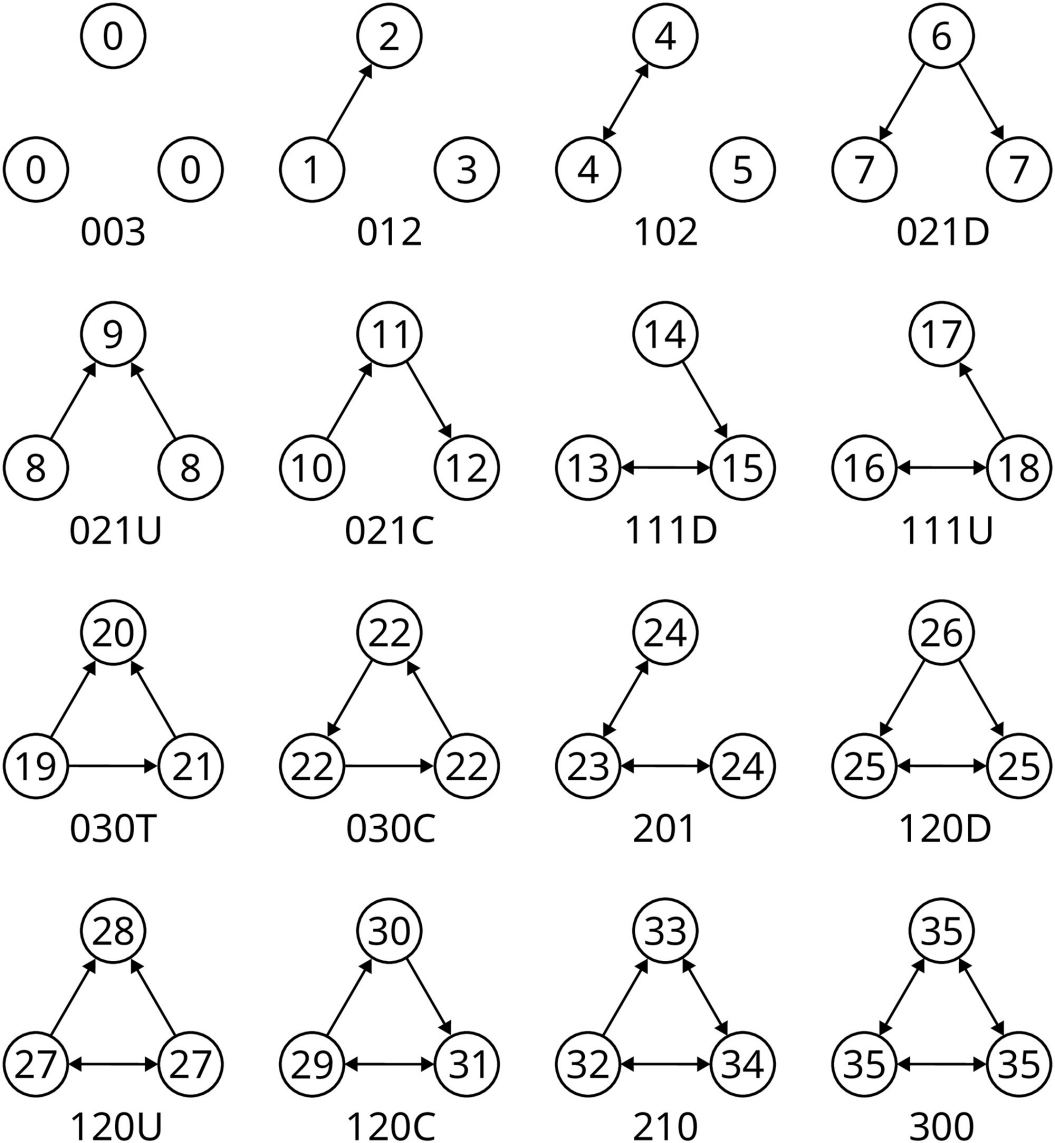
Triads and their associated orbits. Depicition of all 16 possible triads and 36 orbits. Orbits are labeled with integers in nodes. Edges that are present in both directions are represented as a single double arrow.

Consider a directed graph *G* = {*V*, *E*}, where *V* is a set of vertices and *E* is a set of edges, and its undirected graph *G*′ = {*V*, *E*′}. The triadic census represents the count of all possible induced triplets in *G*. Each triad is a separate non-isomorphic subgraph with three nodes. Fig 6 depicts all possible triads and their orbits, i.e. automorphism points. Each triad is defined by its unique label which consists of three digits and a potential character. The first digit represents the number of mutual links, the second digit represents the number of directed links and the third digit represents the number of missing links in a triad. If the arrangement of edges in different triads is the same, the additional characters separate them, where C stands for cycle, T for transitivity, U for up, and D for down [56].

Our algorithm is based on the subquadratic triad census algorithm by Batagelj and Mrvar [57]. It calculates the underlying triads by traversing all connected triplets (*u*, *v*, *w*) in the undirected graph *G*′. Nonetheless, we are only considering connected triads, therefore excluding triads with labels 003, 012, and 102 (see Fig 6). The triad count in this algorithm is updated by the equation

$$\begin{array}{c} \begin{array}{cc} tricode & =Link(v,u)+2*Link(u,v)+4*Link(v,w)+\\ & 8*Link(w,v)+16*Link(u,w)+32*Link(w,u), \end{array} \end{array}$$ (7)

which uniquely maps the triad code to its triad type.

**Fig 7:**
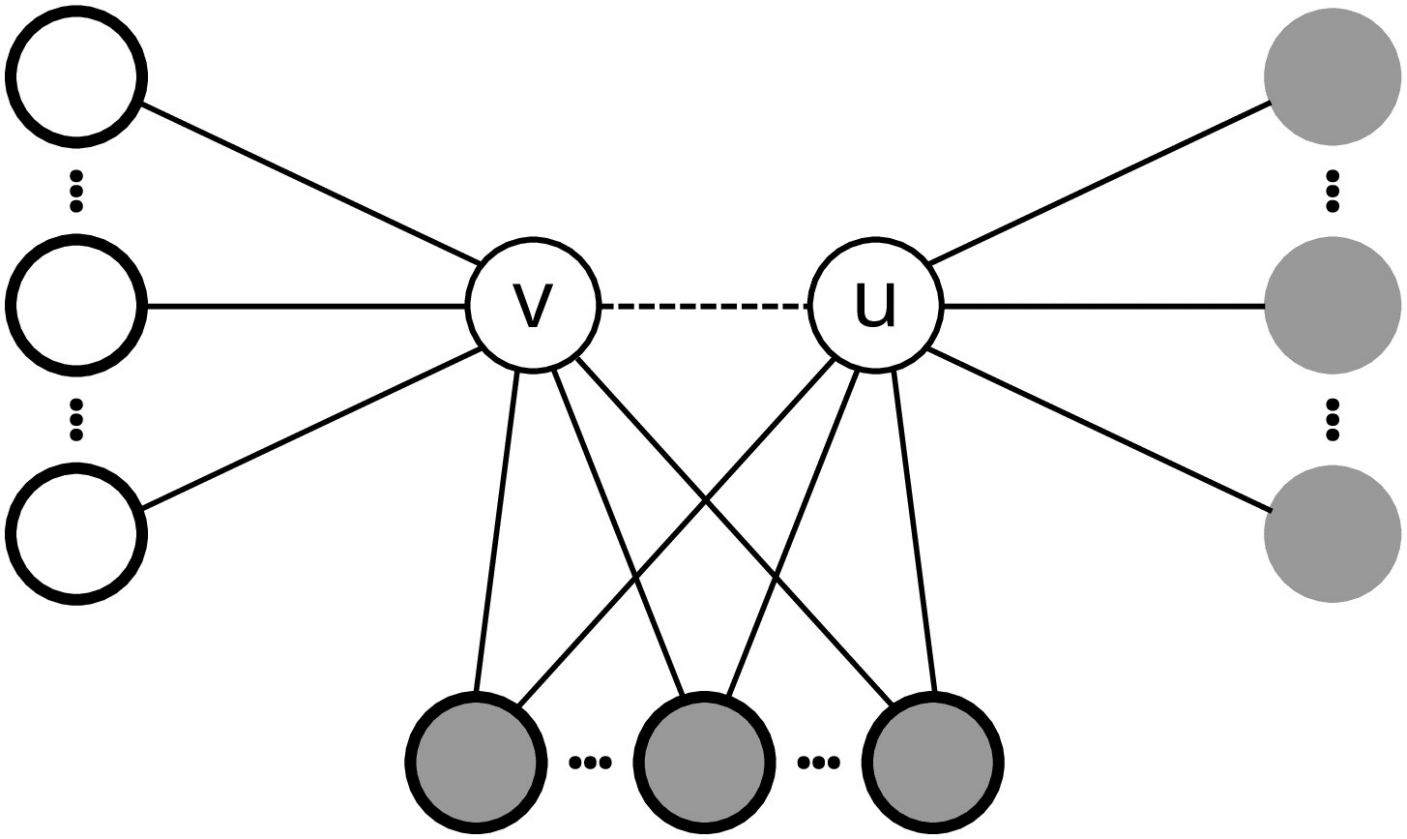
Representation of the induced undirected subgraph. The induced subgraph is defined by nodes *u*, *v* and their neighbors. Nodes with bold outline represent neighbors of *v*, *N*(*v*). Gray nodes represent neighbors of *u*, *N*(*u*). Nodes with gray fill and bold outline are common neighbors of nodes *v* and *u*.

Ortman and Brandes [58] modified the basic algorithm to include the orbit count for each node in *G*, i.e., the count of all automorphism points in a given triad. Similarly, we modified the subquadratic triad census algorithm by quantifying how much each pair of vertices *v* and *u* contributes to the total triad count of *G*. Considering that mutation updates only one edge in the subject’s graph, it is not mandatory to calculate a complete triadic census from scratch. Instead, the update procedure updates the triadic census based on the local neighborhood. If we modify the graph *G* by changing the potential edge (*v*, *u*) by either removing or adding it, then the difference in the triadic census is defined only by the local neighborhoods of nodes *v* and *u*, *N*(*v*) and *N*(*u*) respectively. If edge (*v*, *u*) exists in *G*′ after mutation, then the triadic census is updated based on the triad count of the induced subgraph defined by the set of points {*v*, *u*} ∪ *N*(*v*) ∪ *N*(*u*), otherwise the induced subgraph consisting of points {*v*, *u*} ∪ (*N*(*v*) ∩ *N*(*u*)) is considered (see Fig 7).

The whole procedure consists of two routines Count_triads (see Algorithm 1) and Count_local_triads (see Algorithm 2). In the first step, the whole graph is traversed and the triadic census is calculated with information on how much each pair of vertices contributes to the triadic census. After each mutation (see Algorithm 3), a count on a local neighborhood is performed and the triadic census is accordingly updated. The computational complexity of the subquadratic triad census algorithm is *O*(Δ ⋅ |*E*|), where Δ is the maximum degree of a node in *G*′. The computational complexity of our update procedure is *O*(Δ). Note that by introducing an additional |*V*| × |*V*| × 16 matrix, the space complexity does not change, considering that the adjacency matrix in its raw form requires |*V*| × |*V*| elements.

**Algorithm 1** Count_triads

Input:

*G* = {*V*, *E*} … directed graph

*G*′ = {*V*, *E*′} … underlying undirected graph

Initialization:

census[:] ← 0 … list with 16 elements

pair_count[:, :, :] ← 0 … matrix with |*V*|×|*V*| × 16 elements

**for** *v* ∈ *V* **do**

 *N*(*v*) … neighbors of *v* in *G*′

 **for** *u* ∈ *N*(*v*), where *u* > *v* **do**

  *N*(*u*) … neighbors of *u* in *G*′

  *S* ← *N*(*v*) ∪ *N*(*u*)

  **for** *w* ∈ *S*, where *u* < *w* or (*v* < *w* and not *w* ∈ *N*(*v*)) **do**

   *E*(*a*, *b*) returns 1 if edge exists and 0 otherwise

   tricode ← *E*(*v*, *u*)+ 2*E*(*u*, *v*) + 4*E*(*v*, *w*) + 8*E*(*w*, *v*) + 16*E*(*u*, *w*) + 32*E*(*w*, *u*)

   type ← map tricode to its type

   census[type] = census[type] + 1

   pair_count[*v*, *u*, type] = pair_count[*v*, *u*, type] + 1

   pair_count[*v*, *w*, type] = pair_count[*v*, *w*, type] + 1

   pair_count[*u*, *w*, type] = pair_count[*u*, *w*, type] + 1

  **end for**

 **end for**

**end for**

**return** census, pair_count

**Algorithm 2** Count_local_triads

Input:

*G* = {*V*, *E*} … directed graph

*G*′ = {*V*, *E*′} … underlying undirected graph

*v*, *u* … pair of nodes, where potential edge is modified in *G* and consequently in *G*′

Initialization:

pair_count_local[:] ← 0 … list with 16 elements

*N*(*v*) … neighbors of *v* in *G*′

*N*(*u*) … neighbors of *u* in *G*′

**if** *E*′(*u*, *v*) exists **then**

 *S* ← (*N*(*v*) ∪ *N*(*u*)) − {*v*, *u*}

**else**

 *S* ← (*N*(*v*) ∩ *N*(*u*)) − {*v*, *u*}

**end if**

**for** *w* ∈ *S* **do**

 *E*(*a*, *b*) returns 1 if edge exists and 0 otherwise

 tricode ← *E*(*v*, *u*) + 2*E*(*u*, *v*) + 4*E*(*v*, *w*) + 8*E*(*w*, *v*) + 16*E*(*u*, *w*) + 32*E*(*w*, *u*)

 type ← map tricode to its type

 pair_count_local[type] = pair_count_local[type] + 1

**end for**

**return** pair_count_local

**Algorithm 3** Mutation

Input:

*G* = {*V*, *E*} … directed graph with edges in *E* and missing edges in *E*^*C*^ census … triadic census of *G*

pair_count… matrix with |*V*| × |*V*| × 16 elements

Initialization:

edge_operation ← assign 0 or 1 with the same probability

**if** edge_operation is 1 **then**

 (*v*, *u*) ← randomly select element from *E*^*C*^

 *E* ← *E* ∪ {(*v*, *u*)}

**else**

 (*v*, *u*) ← randomly select element from *E*

 *E* ← *E* − {(*v*, *u*)}

**end if**

pair_count_local ← Count_local_triads(*G*, *v*, *u*)

pair_count_difference ← pair_count_local—pair_count[*v*, *u*, :]—pair_count[*u*, *v*, :]

pair_count[*u*, *v*, :] ← 0

pair_count[*v*, *u*, :] ← pair_count_local

census ← census + pair_count_difference

**return** *G*, *census*

### Selection of the optimal Boolean network

Boolean networks are inferred from the final population of possible underlying directed graphs, i.e. influence networks using the Quine–McCluskey minimization algorithm. Each function is thus represented with its minimal disjunctive normal form. The dynamic error is then assessed for each Boolean network and one with the minimal dynamic error is selected. In case, when multiple Boolean networks have the same minimal dynamic error, the Boolean network closest to the optimal point in normalized objective space is selected. Consider the ideal solution composed of the best extreme solutions in the last Pareto frontier $\bar{z}=\left(z_{1}^{\text{min}},z_{2}^{\text{min}}\right)$, where *z*_1_ is compliance with regulator rankings and *z*_2_ is topological similarity. Values of each axis are normalized to the interval of [0, 1] by the equation

$$\begin{array}{c} z_{i}^{*}=\frac{z_{i}-z_{i}^{\text{min}}}{z_{i}^{\text{max}}-z_{i}^{\text{min}}}. \end{array}$$ (8)

Some regulators may become obsolete and expressions can be reduced to constants by the minimization procedure. In this case, each constant Boolean expression is replaced with a non-constant expression taken from the Boolean network closest to the optimal point in normalized objective space.

The running time of the Quine–McCluskey algorithm grows exponentially with the number of variables, however, SAILoR limits the number of possible regulations while simultaneously following the in-degree distribution of provided reference networks in the process of NSGA-II optimization, further reducing the number of possible regulators. SAILoR thus does not suffer from impractical high computational complexity in the process of minimizing logic expressions. We applied the open-source PyPI package for the Quine–McCluskey minimization available under the MIT License (BSD) [59].

### Hyperparameter tuning and assessment of SAILoR

We evaluated SAILoR for the inference of networks with 16, 32, and 64 nodes. Ten different networks of the same size were first extracted from E. coli GRN and then simulated with the tool GeneNetWeaver [38]. We extracted 10 networks with 16 genes and at least 5 regulators, 10 networks with 32 genes and at least 10 regulators, and 10 networks with 64 genes and at least 20 regulators. Each network was simulated to obtain 10 different time-series gene expression data with 56 time steps. All parameters were the same as in in-silico network challenge DREAM4 [60]. For additional information see [16].

Due to the large number and range of potential parameter values of SAILoR, we performed a hyperparameter tuning procedure using randomized search [61] with 60 iterations for parameters *α*_1_, *α*_2_, *α*_3_, and *α*_4_. Each parameter specifies the weight of a separate topological property in the final topological similarity objective (*z*1). We limited values of alphas to the interval [0, 1] since all topological loss functions take values from the same interval. We repeated 60 iterations of randomized search, which with the 95% probability guarantees that the best configuration found will lie within the top 5% of the optimal area [61].

Parameters *α*_1_, *α*_2_, *α*_3_, and *α*_4_ were assessed on 10 networks with 64 nodes. Within each iteration of the parameter search, we measured the improvement compared to the base network obtained with dynGENIE3 and a prior knowledge of the expected number of regulations. The improvement was measured as an absolute difference between the performance of dynGENIE3 and SAILoR. We assessed improvements for metrics describing static correctness based on the structure of the static underlying directed influence graph. We, therefore, assessed improvements for accuracy, precision, recall, *F*1 score, bookmaker informedness (BM), and the Matthew’s correlation coefficient (MCC). Due to the stochasticity of SAILoR, we repeated the inference for a single network 10 times. As a prior knowledge, we provided structures of all networks with the same size, except the network to be inferred from time-series data. After executing genetic algorithm in SAILoR with 10 generations and 1000 subjects we obtained the following *α* values *α*_1_ = 0.285, *α*_2_ = 0.0604, *α*_3_ = 0.7872, *α*_4_ = 0.3377. Since each initial network was generated based on the expected in-degree distribution, *α*_2_ is understandably low, however, the high value of *α*_3_ indicates the significance of the triadic census when considering topological properties.

We assessed the ability of SAILoR to infer static graphs on all networks with the obtained *α* values. We measured the improvement of structural correctness of the candidate network closest to the ideal network in normalized objective space. The optimal point in normalized space is ${\bar{z}}^{*}=\left(0,0\right)$ and the solution closest to the ideal point is

$$\begin{array}{c} \left({\tilde{z}}_{1}^{*},{\tilde{z}}_{2}^{*}\right)=\underset{\left(z_{1}^{*},z_{2}^{*}\right)}{\text{arg}\;\text{min}}\sqrt{z_{1}^{*2}+z_{2}^{*2}}. \end{array}$$ (9)

The optimal point in the normalized objective space represents the best compromise between the topological similarity to the reference networks and the alignment with time-series gene expression data. While this does not automatically guarantee the improvement of static accuracy compared to dynGENIE3, our results indicate that the improvement is significant in the majority of cases for various network sizes (see section Structural properties of inferred networks). We provided all other networks of the same size as a reference and ran SAILoR for 10 generations with 1000 candidate networks.

Rarely, however, will the network closest to the optimal point in normalized objective space have the best dynamic accuracy. To evaluate the capacity of SAILoR to infer accurate Boolean networks, we measured the dynamic as well as the static performance with 10-fold cross-validation and averaged the obtained results across all networks of the same size (see section Dynamic properties of inferred networks). In each cross-validation iteration, one time-series experiment was excluded in the process of network inference and utilized in the process of validation. Each network was inferred from a 9 time series with 56 time steps, which we merged into a single time series with 9 ⋅ 56 time steps. Each network was evaluated on the excluded time series. We provided all other networks of the same size as a reference and ran SAILoR for 10 generations with 1000 candidate networks.

## Results and discussion

### Structural properties of inferred networks

**Fig 8:**
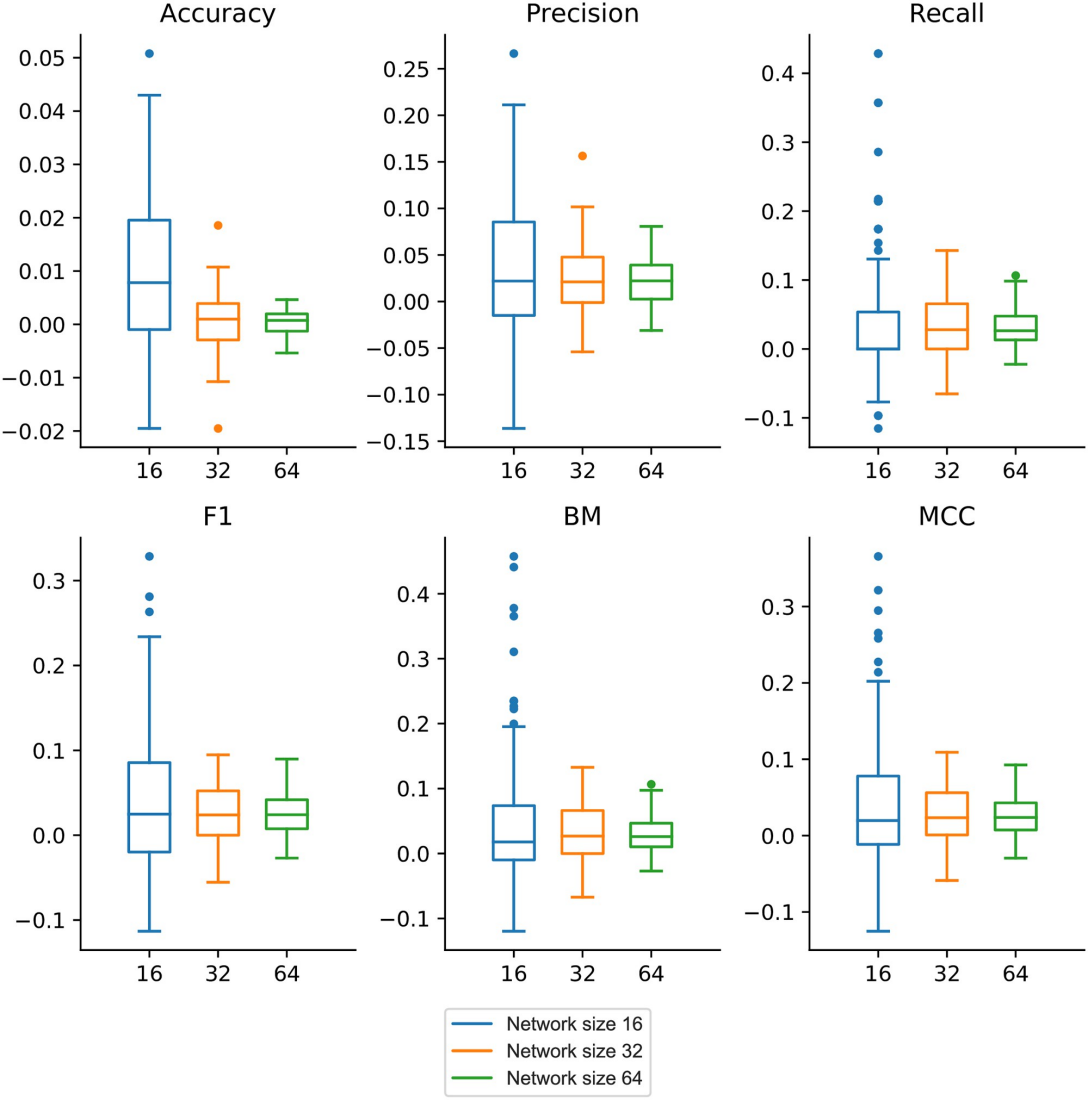
Performance improvements of SAILoR compared to dynGENIE3. Accuracy, Precision, Recall, *F*1 score, Bookmaker informedness (BM), and Matthew’s correlation coefficient (MCC) improvements are calculated as a difference between the performance of SAILoR and the dynGENIE3 method. Blue boxes indicate improvements for networks with 16 nodes, orange boxes denote improvements for 32 node networks, and green boxes represent 64 node network improvements. Results are obtained from 10 repetitions of 10 different networks for each network size.

In comparison to dynGENIE3, the incorporation of prior knowledge in terms of expected network topology significantly improves the correctness of inferred static networks in the majority of cases for all the observed metrics, except for the static accuracy for networks with 32 and 64 nodes (see Fig 8). This indicates that SAILoR compensates for the correct prediction of non-edges to increase the topological similarity, which results in a higher ratio of correctly identified edges (see Precision, Recall and *F*1 score in Fig 8). However, due to the overall sparsity of biological GRNs, the number of missing edges is much larger than the number of edges. Therefore, the identification of true edges bears a higher importance than the identification of missing edges. The improvement of Bookmaker informedness (BM) and Matthew’s correlation coefficient (MCC) indicate that SAILoR has higher predictive power than the dynGENIE3 since both metrics compare the classifier performance with random guessing (see BM and MCC in Fig 8).

### Dynamic properties of inferred networks

**Fig 9:**
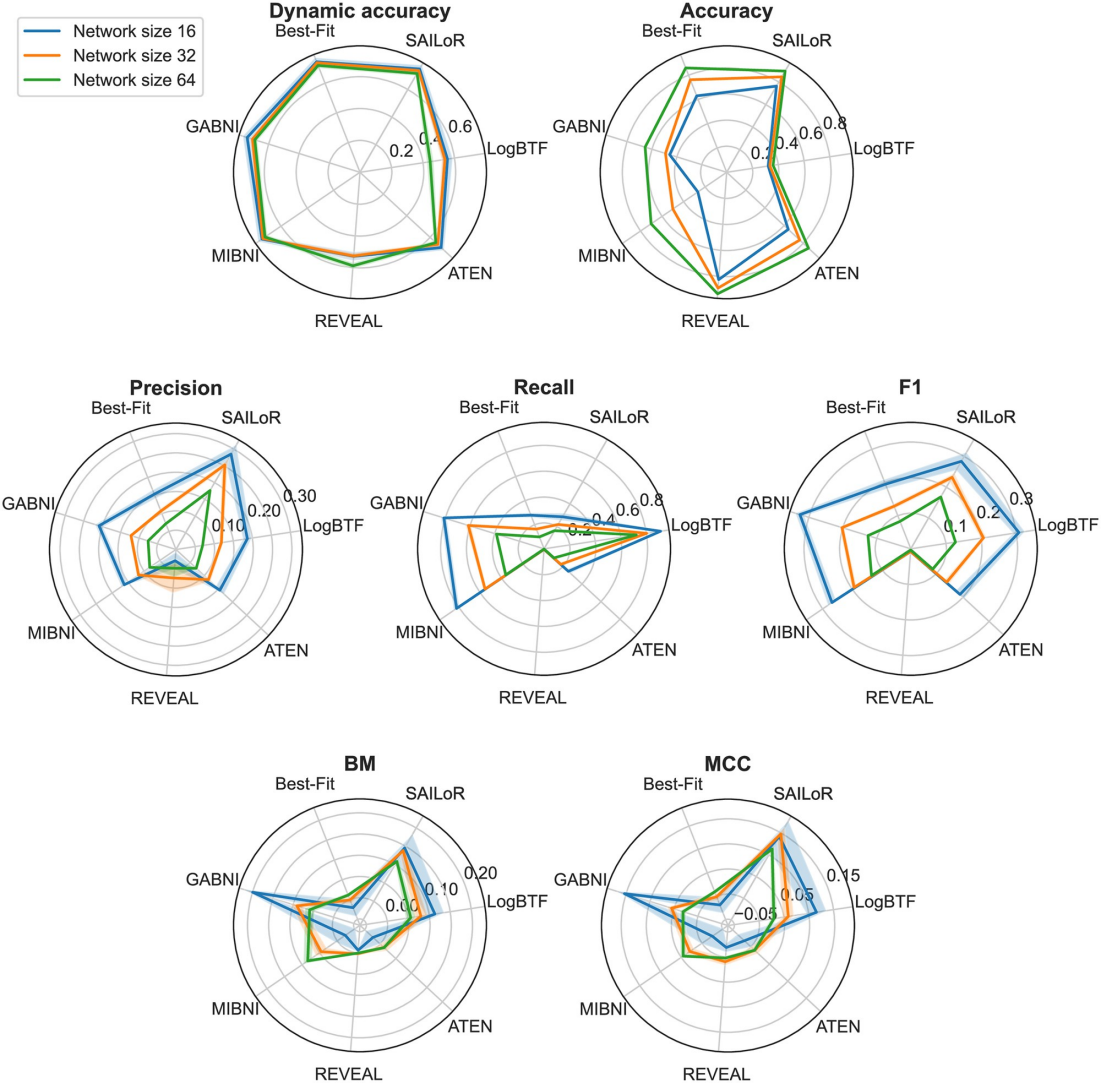
Performance of Boolean inference methods. Performance of Boolean inference methods in terms of dynamic accuracy, static accuracy, precision, recall, *F*1, bookmaker informedness (BM), and Matthew’s correlation coefficient (MCC). Solid lines denote mean values and shaded areas represent 95% confidence intervals.

We compared the performance of SAILoR with the performance of other Boolean network inference approaches, namely Best-Fit [15], REVEAL [7], MIBNI [8], GABNI [5], ATEN [4], and LogBTF [10]. Our results show that SAILoR retained the ability to infer Boolean networks with high dynamic accuracy while improving the correctness of the underlying influence network (see Fig 9). Overall SAILoR has the 2nd highest accuracy, right behind REVEAL, however, since REVEAL infers regulation rules where no inconsistencies are present, the number of inferred regulations is often small. For this reason, REVEAL suffers from low dynamic accuracy and very low precision, recall, and *F*1 score.

Both SAILoR and LogBTF achieve high scores for bookmaker informedness and Matthew’s correlation coefficient. However, the dynamic performance of LogBTF on our dataset is low, for which we hypothesize several reasons. First, Boolean threshold functions may not be as suitable for the representation of GRNs as disjunctive normal forms since Boolean threshold functions cannot describe an arbitrary logic function. For example, exclusive or, and logical equivalence cannot be represented with a Boolean threshold function. Second, LogBTF seems to be more sensitive to errors in our dataset, which arose from merging multiple time series data into a single time course. Errors often occur in real experimental data due to noisy conditions and measurement errors [62]. Finally, Li et al. [10] evaluated the dynamic performance of LogBTF on the training data, while 10-fold cross-validation was used in our case. This may additionally explain the discrepancy between our results and the results reported by Li et al.

**Fig 10:**
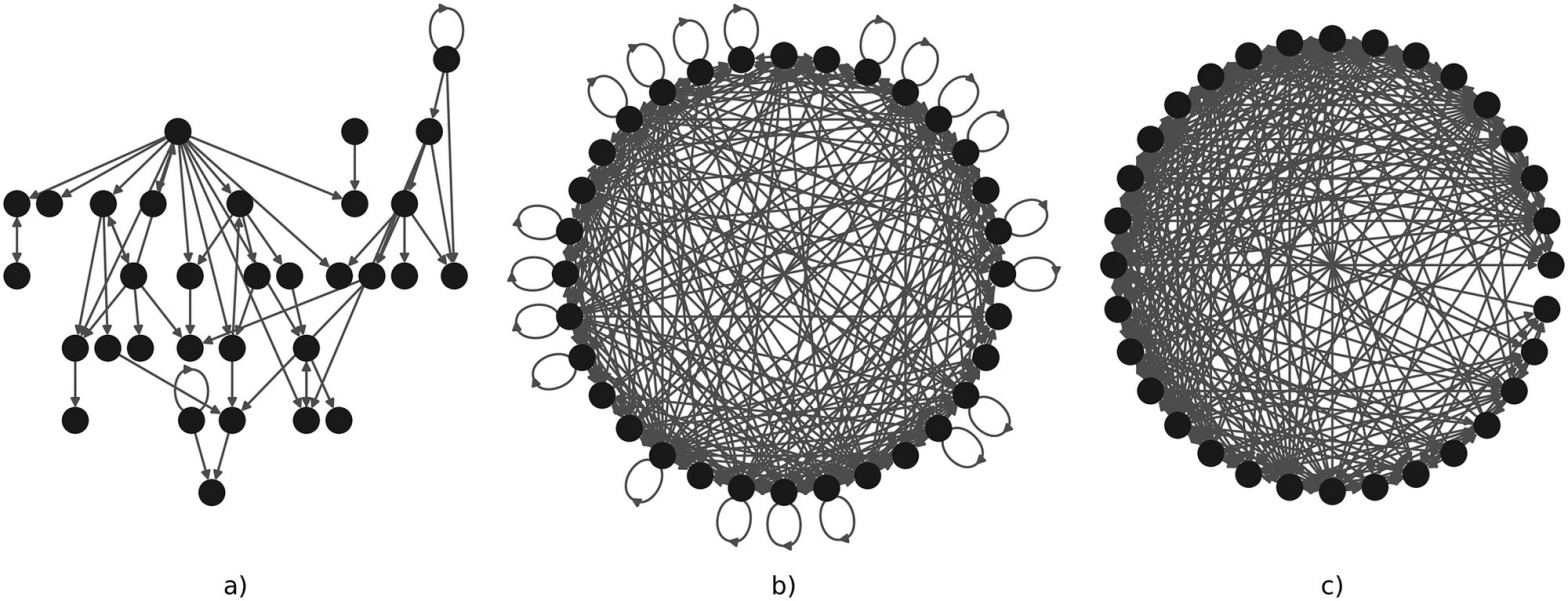
Example of Boolean networks’ influence graphs obtained with SAILoR, MIBNI, and GABNI. Fig a), b) and c) illustrate the influence graphs of the Boolean networks with 32 nodes obtained with the inference methods SAILoR, MIBNI and GABNI, respectively.

**Fig 11:**
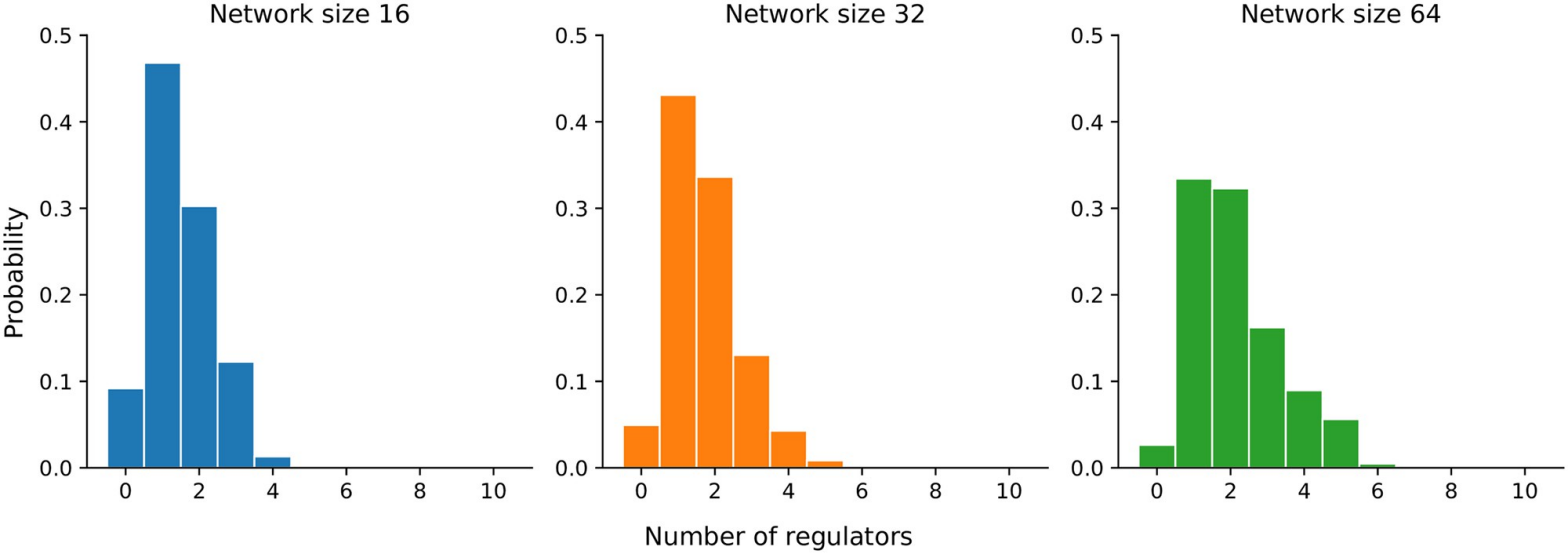
In-degree distributions of Boolean functions inferred with SAILoR. In-degree distributions of inferred Boolean functions are extracted from all evaluated networks with 16, 32, and 64 nodes. The vertical axis displays the probability of a Boolean function consisting of multiple distinct regulators. Bin colors represent different network sizes.

The bookmaker informedness score of SAILoR is right behind the score of GABNI in terms of inferring small networks. Both MIBNI and GABNI suffer from low static accuracy, as both methods overestimate the number of regulators in Boolean functions in order to achieve higher dynamic accuracy. Nevertheless, in most cases, such networks are overfitted to the provided binarized time-series data. While MIBNI limits the number of regulators to 10, the upper bound of regulators per node in GABNI is 0.6*n*, where *n* is the number of nodes in a network. Such networks are not biologically relevant, since they overestimate the number of regulators (see Fig 10). Fig 11 displays the in-degree distributions of Boolean functions inferred with SAILoR, which represent the probability of a logic function with a certain number of inputs. The distributions are separated according to the network size, whereby all logic functions from evaluated networks are taken into account. Boolean functions of evaluated networks derived with SAILoR consist mainly of at most 5 regulators. The in-degree distribution is slightly more skewed to the right for logic functions from networks with 64 nodes, however, only 0.4% (25 of 6, 400) of such Boolean functions have seven or more distinct inputs. This shows, that constraining the number of regulators to 10 has no impact on the dynamic accuracy of networks inferred with SAILoR. SAILoR has a high precision and a high *F*1 score, while the recall is low. This indicates that SAILoR infers a lower number of regulations, while it simultaneously increases the ratio of all correct edges among all predicted edges. Overall, Boolean networks inferred with SAILoR are biologically more relevant and have high dynamic accuracy and high structural correctness.

### Computational complexity

The computational complexity of SAILoR depends on multiple factors. The main contributors to the overall running time are the number of generations (*G*), the number of subjects (*N*), the problem size, i.e. number of nodes in the inferred network (*n*), and the total number of time points (*T*). Under the assumption that the size of the provided reference networks is of the same order as the problem size, the computational complexity for extraction of topological properties from reference networks is *O*(*m*(3*n*^2^ + Δ|*E*|)), where *m* is the number of networks and |*E*| is the expected number of edges. In the worst case, the maximum degree in a network (Δ) is *n*. In addition, we can approximate the number of edges in a scale-free network to

$$\begin{array}{c} \left|E\right|\approx n\sum_{k=1}^{n}\frac{1}{k^{\lambda -1}}⪅n\sum_{k=1}^{\infty }\frac{1}{k^{\lambda -1}}⪅nC, \end{array}$$ (10)

where *C* is a constant. Note that for the typical values of λ (2 < λ < 3) [41] the above series (Eq 10) converges [63]. Our experiments indicate sufficient performance of SAILoR for the constant number of reference networks, regardless of the problem size. Under the above assumptions, the computational complexity for extraction of topological properties can be reduced to *O*(*n*^2^). Computational complexity of dynGENIE3 is *O*(*n*^2^*TlogT*) [20]. Both methods are executed only once. Furthermore, the computational complexity of the fast non-dominated sorting in SAILoR is *O*(2*N*^2^) [35] given a fixed number of optimization objectives. The mutation of each subject has a complexity of *O*(*n*^2^), however, the update procedure of topological properties after the mutation has *O*(1 + *n*) complexity. The update of network density and degree distributions is performed in constant time, whereas the triadic census update takes *O*(*n*) time. The computational complexity of crossover is *O*(*n*^2^). In addition, all topological properties need to be recalculated, which can be done in *O*(4*n*^2^) time. Overall, the computational complexity of GA in SAILoR, including the calculation of subjects’ initial topological properties, is *O*(*Nn*^2^ + *G*(*Nn*^2^ + *N*^2^)), which is reduced to *O*(*GNn*^2^). In the final step, SAILoR performs iterative k-means binarization [14], which can be computed in *O*(*nT*) time, considering the constant number of re-clustering iterations and the constant iterative depth of 3. The inference of a Boolean network from a given adjacency matrix with the Quine–McCluskey algorithm is bounded by $O(r^{2}3^{r})$ running time, where *r* is the number of regulators [64]. For a constant number of regulators, this computational complexity is constant. While this constant is not negligible for large *r*, we must note, that a very small fraction of nodes have a high number of regulators in biological networks [47]. Furthermore, the number of regulators for each target gene in SAILoR is constrained to 10. At the final step, every Boolean network is evaluated at *T* time points. This can be done in *O*(*nNT*) time. The overall computational of SAILoR is *O*(*n*^2^*TlogT* + *GNn*^2^ + *nNT*).

We have parallelized SAILoR in multiple stages to reduce its running time. First, ranked interactions are obtained with parallel execution of dynGENIE3. In addition, the inference of Boolean rules and the identification of a suitable Boolean network are executed in parallel. The DEAP library allows simultaneous evaluation of subjects, however, in our case the sequential execution of the genetic algorithm proved to be less time-consuming, due to the communication overhead between different processes. Communication overhead is especially prominent in Python since the global interpreter lock (GIL) prevents parallel execution of multiple threads [65]. SAILoR required on average approximately 7 minutes and 41 seconds to infer a Boolean network with 64 nodes on 32 allocated AMD EPYC 7453 CPU cores.

### Inference of context-specific gene regulatory subnetworks of *Drosophila melanogaster*

To demonstrate the applicability of SAILoR we inferred context-specific Boolean networks describing the genetic response of Drosophila melanogaster to mating. Delbare et al. [39] measured female neural gene expression profiles for 9, 027 expressed genes of D. melanogaster at 0.5, 1, 2, 3, 4, 5, 6, 8, 12 and 24 hours after mating and for virgin flies. The authors show that the seminal fluid protein, i.e. Sex peptide (SP) alters a female’s circadian rhythm after mating, which may play a role in a virgin-to-mated transcriptional switch and in changes of metabolic gene regulatory network.

Due to the large gap in measurements between 8, 12, and 24 hours, we considered measurements from 1 to 8 hours and interpolated gene expressions at 7 hours after mating. We are interested in the Boolean dynamics between genes, whose role is directly associated with egg-laying behavior, regulation of female receptivity, and circadian behavior. We therefore observed *frma, per, egh, na, Est-6, IBIN, Clk, NPF, SPR, cyc, JhI-26, tim, spin, mbl, pain, dati, Rack1, slo, ash1, cry, lectin-46Cb, loj, sra, Rh7, Ide, Sk2, Tdc2, Esp, Gadd45, SIFa, Tmc, Pdfr, retn, hdly, Nrg, BTBD9*, and *CG10433*. Each time-series CPM (count per million) data is available for three independent biological replicates. Nonetheless, we discarded the last replicate due to missing data.

Furthermore, we obtained the reference network of female Drosophila constructed with the context-specific inference method NetREX [36]. The initial network contains 300, 000 ranked interactions for 7, 530 genes. We selected the top 60, 000 interactions and extracted 10 sub-networks with 37 nodes and at least 20 regulators. We inferred the context-specific Boolean network of D. melanogaster after mating (M), and the Boolean network of a virgin (V) D. melanogaster from CPM measurements.

**Fig 12:**
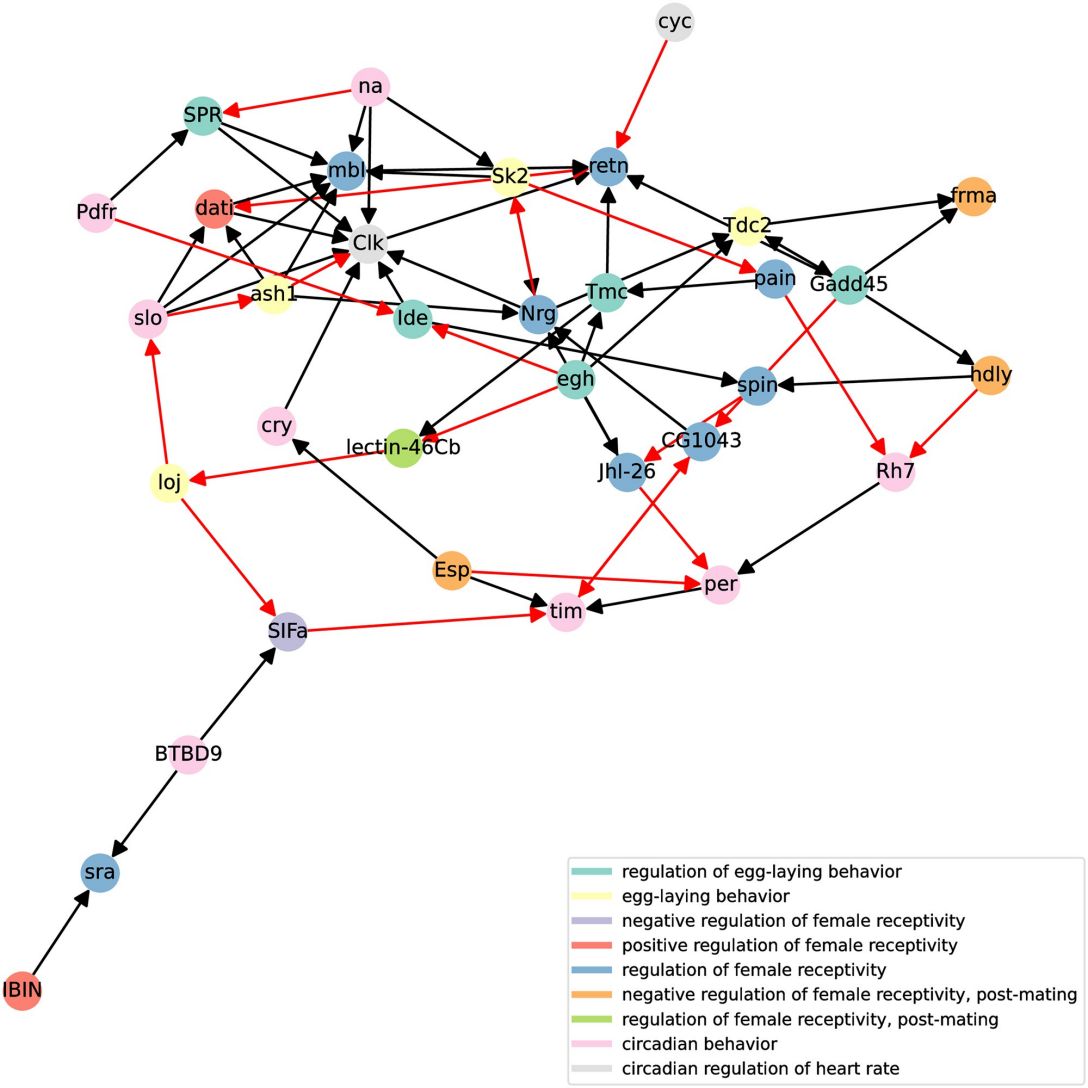
Influence graph of a context-specific Boolean network of virgin female *Drosophila melanogaster*. Static representation of the virgin Boolean network (V). Black and red edges represent positive and negative influences, respectively. Each node is colored according to its known biological role.

**Fig 13:**
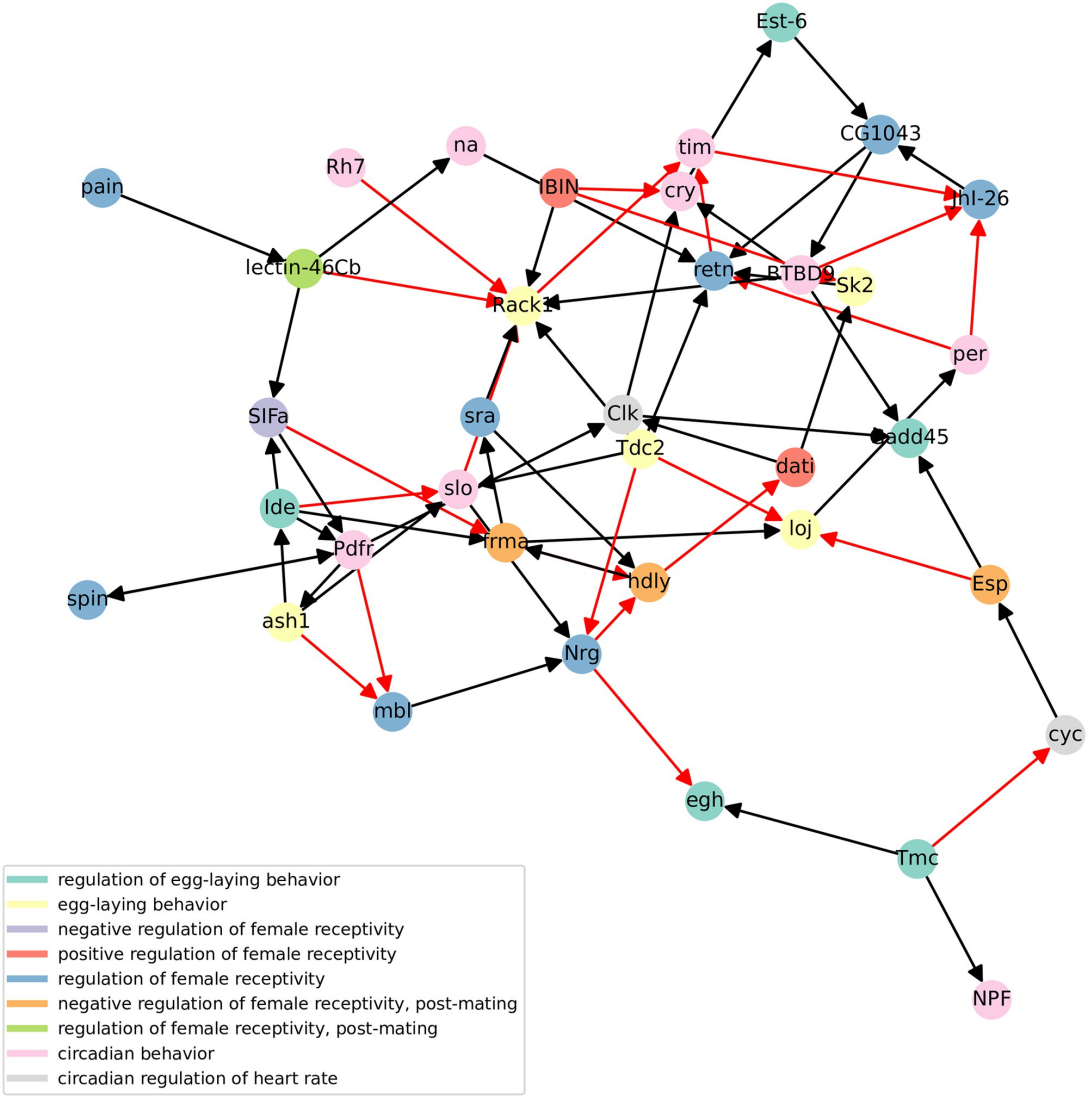
Influence graph of a context-specific Boolean network of female *Drosophila melanogaster* after mating. Static representation of the mating Boolean network (M). Black and red edges represent positive and negative influences, respectively. Each node is colored according to its known biological role.

The dynamic accuracy of (V) Boolean network is 0.66 for the first time series and 0.77 for the second time series, while the dynamic accuracy of (M) network is 0.68 for the first time series and 0.73 for the second time series. Obtained dynamic accuracy is in the expected range according to Fig 9. Nonetheless, we must note that SAILoR does not overfit Boolean network to binarized time-series data, since it also considers continuous data as well as given structural constraints. However, if we apply (V) time-series data to (M) Boolean network, the dynamic accuracy falls to 0.56 and 0.52. Similarly, if we apply (M) time-series data to (V) network, the dynamic accuracy falls to 0.55 for both replicates, which is slightly above random guessing. This further demonstrates the need to infer context-specific dynamical models of biological systems. Figs 12 and 13 display static representation, i.e. influence graphs, of Boolean networks (V) and (M) respectively. We assessed the positive and negative influence of connections with truth tables. If the target gene is predominantly the same as its regulator, then we assigned positive influence. Otherwise, we marked the edge as a negative influence. Due to the small subnetwork size, each edge can depict direct or indirect interaction. In addition, some edges may be fabricated by SAILoR to best fit the given constraints and a Boolean model.

Both networks differ in many interactions, signifying the importance of a proper context when inferring Boolean networks. For example, consider the positive interaction of Clk and cry from the network (V) (see Fig 12). Protein CRY has been linked to circadian rhythmicity as a blue light photoreceptor dedicated to mediating TIM degradation [66] and to resetting of circadian rhythms [67]. However, in the network (M) this interaction is reversed (see Fig 13). This further indicates that the mating disrupts the circadian rhythm of Drosophila. While the known negative regulation of tim by cry is missing from both networks, SAILoR still identified indirect positive regulation of Clk through double repression, since TIM-PER heterodimer inhibits CLK-CYC activity. We must also note, that SAILoR unsuccessfully identified the linked role of Clk and cyc in the downstream regulation of other clock genes [68]. For example, CLK and CYC directly activate transcription of per and tim [66]. Nonetheless, SAILoR still identified the combined role of CLK-CYC dimer through joint regulation of retn in (V) network and through the indirect regulation of Gadd45 in (M) network. The Dead ringer protein (RETN) is implicated as a major repressor of male courtship behavior [69]. In (M) network retn is regulated by per. Additionally, RACK1, an essential receptor at multiple steps of Drosophila development, particularly in oogenesis [70], is in the network (M) heavily regulated. However, Rack1 was reduced to a constant 1 in network (V). While the relationship between the molecular clock genes and the regulation of female receptivity and regulation of egg-laying behavior has not been yet completely explained, it has been shown, that the altered circadian expression impacts metabolic and neuronal features [39].

## Conclusions

SAILoR infers Boolean representations of gene regulatory networks with the utilization of time-series gene expression data and incorporation of prior knowledge of the structure of the inferred network. We performed a rigorous evaluation of SAILoR. First, we assessed the ability of SAILoR to infer accurate static networks, which act as influence graphs for Boolean networks. We evaluated SAILoR on subnetworks extracted from the E. coli. Our results indicate a significant improvement in static accuracy to the method dynGENIE3 for the majority of cases (see section Structural properties of inferred networks). In addition, we evaluated the ability of SAILoR to infer Boolean networks. We compared the performance of SAILoR with other Boolean network inference approaches including Best-Fit, REVEAL, MIBNI, GABNI, ATEN, and LogBTF. We demonstrated that the incorporation of prior knowledge in terms of general network structure allows SAILoR to improve the structural correctness of inferred Boolean networks while maintaining dynamic accuracy (see section Dynamic properties of inferred networks). Furthermore, we utilized SAILoR to infer the context-specific Boolean subnetworks of Drosophila melanogaster before and after mating (see [39]). Obtained networks differed in numerous interactions, which in our opinion demonstrates the necessity for context-specific dynamical models.

Due to large space and time complexity, the current implementation of SAILoR is impractical for larger problems. This problem could be mitigated with the utilization of sparse matrices, however, in this case, the time complexity of SAILoR would increase, due to additional operations specific to sparse matrices. For this reason, SAILoR is appropriate for the inference of small to medium-sized Boolean networks. Nonetheless, even larger networks could be inferred with the aid of SAILoR. For example, one could infer a Boolean network consisting of known regulators using SAILoR and additionally apply, for example, Best-Fit to infer logical functions of the remaining genes. Best-Fit is a bit faster and not as memory-consuming as SAILoR. Best-Fit could be additionally modified to infer logic functions from a reduced set of potential regulators obtained for example with the application of the mutual information-based feature selection as used in MIBNI. Nonetheless, SAILoR can still be applied in the presented form to infer specific subsystems of real-world gene regulatory networks. We demonstrated this with the inference of context-specific Boolean networks describing the dynamics of virgin D. melanogaster and the dynamics of D. melanogaster after mating.

Another limitation of SAILoR is that the size of reference networks should be of the same order as the network being inferred. Currently, SAILoR utilizes expected network density as one of its objectives. This property is proportional to the squared number of nodes in the networks. The network density differs between networks of various sizes that have the same number of expected edges per node. To consider reference networks of different sizes, SAILoR could be modified to take into account the expected number of edges per node. Instead, we argue that in most cases reference networks will and should be of approximately the same size.

Nonetheless, SAILoR offers numerous advantages to other assessed methods for inference of Boolean networks. SAILoR predicts a Boolean network from both continuous and binarized time series data. For this reason, inferred interactions better align with the underlying static representation of the GRN process. In addition, SAILoR does not overestimate the number of regulators that form a Boolean function since it considers the topological properties of reference networks. In our experiments, we constrained the number of regulators of a gene to 10. However, this value could be revisited when inferring larger Boolean models. SAILoR maneuvers between multiple objectives to infer biologically relevant Boolean networks, which are not overfitted to the binarized time series data. SAILoR could be therefore applied, to infer and predict the dynamics of context-specific biological sub-systems, which we believe is an important step also towards the reconstruction of personalized models for personalized medicine applications.
