# Correction: SAILoR: Structure-aware inference of logic rules

**DOI:** 10.1371/journal.pone.0319814

**Published:** 2025-03-05

**Authors:** Žiga Pušnik, Miha Mraz, Nikolaj Zimic, Miha Moškon

In the Optimization objectives subsection of the Materials and methods, there is an error in the equation three. Please view the complete, correct equation here:


$$z_{1}\left(\hat{x}\right)=L\left(\hat{x},x\right)=\frac{2\sum_{e\in E}\;rank\left(e\right)}{\left|E\right|+{\left|E\right|}^{2}}-\frac{2\sum_{m\in E^{C}}\;rank\left(m\right)}{\left|E^{C}\right|+\left|E^{C}\right|{\left|V\right|}^{2}+\left|E^{C}\right|\left|E\right|'}$$
