# Supplementary material for: SAILoR: Structure-Aware Inference of Logic Rules
Source: PLoS One. 2024 Jun 11;19(6):e0304102. doi: 10.1371/journal.pone.0304102 (PMC11166287; doi:10.1371/journal.pone.0304102)
Supplement: Attachment — Submitted filename: PLoS_One_response.pdf [file pone.0304102.s001.pdf]

Response to the Editor and the Reviewers

Firstly, we would like to sincerely thank the editor and the reviewers for the time invested in reviewing the manuscript and providing insights to enhance the quality of our paper. We addressed all the concerns raised by the editor and the reviewers and tried to follow all their suggestions as much as possible.

Detailed responses to comments are given below. Our revision now also includes the revised manuscript in which all the modifications are marked.

Note: Reviewers' comments are colored red. Responses and specific modifications/edits are colored black.

Editor:

General comments:

Thank you for submitting your manuscript to PLOS ONE. After careful consideration, we feel that it has merit but does not fully meet PLOS ONE's publication criteria as it currently stands. Therefore, we invite you to submit a revised version of the manuscript that addresses the points raised during the review process. Kindly refer to the comments and suggestions below from the reviewers to improve the manuscript before resubmission.

Response:

Thank you for your comments. We addressed all the concerns raised by the reviewers to make the manuscript suitable for publication in PLOS ONE. We also made several additional modifications to improve the quality of our work. Here we provide a broad outline of the changes to the manuscript, which are later explained in more detail.

- We added additional examples to demonstrate the applied genetic operators.
- In our comparison, we now include the additional method called LogBTF to provide a comparison of SAILoR with a more recent method for inference of Boolean networks.
- We excluded the comparison of running times since these results can be misleading due to their potential bias and dependence on the availability of the HPC resources. Variation of running times can thus lead to wrong conclusions in the interpretation of the obtained results.
- We reran SAILoR to eliminate biases, which arose from different treatment of multiple time series data. Since not all Boolean inference methods support learning from multiple time series data, we merged multiple time series into a single one. We, therefore, reran SAILoR on the same combined time series data as all other methods. New results are unbiased since all methods are tested on the same dataset, excluding continuous and prior knowledge data.
- We analyzed the in-degree distributions of Boolean functions inferred with SAILoR. We thus demonstrated that a negligible fraction of functions describing GRN interactions contain seven or more distinct regulators, while the majority of functions consist of 5 regulators or less.

Journal Requirements:

Comment #1:

Please ensure that your manuscript meets PLOS ONE's style requirements, including those for file naming. The PLOS ONE style templates can be found at

https://journals.plos.org/plosone/s/file?id=wjVg/PLOSOne_formatting_sample_main_body.pdf and

https://journals.plos.org/plosone/s/file?id=ba62/PLOSOne_formatting_sample_title_authors_affiliations.pdf

Response:

Thank you for providing additional PLOS ONE style templates. We corrected image references from "Fig." to "Fig" and equation references to "Eq". We accordingly renamed all figure files.

Comment #2:

Thank you for stating the following financial disclosure:

"The research was partially supported by the scientific-research program P2-0359 and by the basic research project J1-50024, both financed by the Slovenian Research and Innovation Agency. The research was also supported by the infrastructure program ELIXIR-SI RI-SI-2 financed by the European Regional Development Fund and by the Ministry of Higher Education, Science and Innovation of the Republic of Slovenia."

Please state what role the funders took in the study. If the funders had no role, please state: "The funders had no role in study design, data collection and analysis, decision to publish, or preparation of the manuscript."

If this statement is not correct you must amend it as needed.

Please include this amended Role of Funder statement in your cover letter; we will change the online submission form on your behalf.

Response:

We would like to clarify that the funders had no role in the study design, data collection and analysis, decision to publish, or preparation of the manuscript.

As instructed, we included the Role of the Funder statement in the cover letter.

Reviewer #1:

General comments:

The study presents a method to infer gene regulatory systems described by Boolean networks.
It is generally well-written, except for some minor grammatical improvements on the abstract and typographical , so that it would be appreciated more by readers.

Response:

Thank you for your positive comments. We have thoroughly revised the manuscript to correct grammatical and typographical errors, and improved phraseology.

Comment #1:

How did you arrive at the limit of the number if regulators to at most 10? The authors have mentioned that this is justified by the scale-free property of networks, but it is not intuitive. Please include the explanation in the manuscript.

Response:

Thank you for your comment. In our manuscript, we included additional justification, where we refer to recent reviews and analyses of Boolean biological models. These reviews show that a negligible fraction of logic functions consist of 10 or more regulators. For example, Mitra et al. analyzed three datasets of curated Boolean models in which only 60 out of 8,871 Boolean functions have more than 10 inputs. In addition, we analyzed the in-degree distribution of logic functions inferred with SAILoR (see Fig 11). Boolean functions of evaluated networks derived with SAILoR consist mainly of at most 5 regulators. The in-degree distribution is slightly more skewed to the right for logic functions from networks with 64 nodes, however, only 0.4% (25 of 6,400) of such Boolean functions have seven or more distinct inputs.

Comment #2:

Were the results be different (ignoring the computational overhead) if the threshold number of regulators is different from 10?

Response:

Our findings indicate that constraining the number of regulators to 10 has no impact on the dynamic accuracy of networks inferred with SAILoR. However, this value could be revisited when inferring larger Boolean models, as we explained in the conclusion.

Comment #3

Have you checked various sources re linked role of clk and cyc in the downstream regulation of other clock genes? Note that reliability of regulation must also be checked.

Response:

Thank you for your comment. Based on your observations, we extended Section 4.4 in our manuscript.

“Both networks differ in many interactions, signifying the importance of a proper context when inferring Boolean networks. For example, consider the positive interaction of Clk and cry from the network (V) (see Fig 12). Protein CRY has been linked to circadian rhythmicity as a blue light photoreceptor dedicated to mediating TIM degradation [66] and to resetting of circadian rhythms [67]. However, in the network (M) this interaction is reversed (see Fig 13). This further indicates that the mating disrupts the circadian rhythm of Drosophila. While the known negative regulation of tim by cry is missing from both networks, SAILoR still identified indirect positive regulation of Clk through double repression, since TIM-PER heterodimer inhibits CLK-CYC activity. We must also note, that SAILoR unsuccessfully identified the linked role of Clk and cyc in the downstream regulation of other clock genes [68]. For example, CLK and CYC directly activate transcription of per and tim [66]. Nonetheless, SAILoR still identified the combined role of CLK-CYC dimer through joint regulation of retn in (V) network and through the indirect regulation of Gadd45 in (M) network. The Dead ringer protein (RETN) is implicated as a major repressor of male courtship behavior [69]. In (M) network retn is regulated by per. Additionally, RACK1, an essential receptor at multiple steps of Drosophila development, particularly in oogenesis [70], is in the network (M) heavily regulated. However, Rack1 was reduced to a constant 1 in network (V). While the relationship between the molecular clock genes and the regulation of female receptivity and regulation of egg-laying behavior has not been yet completely explained, it has been shown, that the altered circadian expression impacts metabolic and neuronal features [39].”

Reviewer #2:

General comments:

The propsed method names as SAILOR uses time-series gene expression data and incorporates prior knowledge of network structure to infer Boolean representations of gene regulatory networks (GRNs). However, SAILoR's current implementation is impractical for larger problems due to space and time complexity. It is best suited for small to medium-sized networks but could be extended with the aid of other methods for larger networks. A limitation is that reference networks should be of similar size to the network being inferred. The paper is well-organized, starting with an introduction of the key challenges, then providing necessary background, and describing the approach and experiments in detail. The writing clearly explains the techniques and results. Relevant work is cited when introducing existing methods and concepts, positioning their contributions in the context of the state-of-the-art. Despite this, I believe that there are several aspects that must be reviewed before the work is acceptable for publication

Response:

Thank you for your review and for your encouraging comments, which we have thoroughly attended to.

Comment #1:

The use of Boolean network is a key aspect of this work. However, the state of the art on this topic is not analyzed in depth in the introduction.

Response:

Thank you for your comment. We extended the introduction and included additional references, namely

- Liu, Xiang, et al. "GAPORE: Boolean network inference using a genetic algorithm with novel polynomial representation and encoding scheme." *Knowledge-Based Systems* 228 (2021): 107277.
- Li, Lingyu, et al. "LogBTF: gene regulatory network inference using Boolean threshold network model from single-cell gene expression data." *Bioinformatics* 39.5 (2023): btad256.
- Weidner, Felix M., et al. "GatekeepR: an R Shiny application for the identification of nodes with high dynamic impact in Boolean networks." *Bioinformatics* 40.1 (2024): btae007.
- Kadelka, Claus, et al. "A meta-analysis of Boolean network models reveals design principles of gene regulatory networks." *Science Advances* 10.2 (2024): eadj0822.
- Mitra, Suchetana, et al. "Preponderance of generalized chain functions in reconstructed Boolean models of biological networks." *Scientific Reports* 14.1 (2024): 6734.

Comment #2:

Moreover, since there are various models to solve GRN, I suggest to the authors to review some recent methods like:

• Pirgazi, Jamshid, Mohammad Hossein Olyaee, and Alireza Khanteymoori. "KFGRNI: A robust method to inference gene regulatory network from time-course gene data based on ensemble Kalman filter." Journal of Bioinformatics and Computational Biology 19.02 (2021): 2150002.

• Seçilmiş, Deniz, Thomas Hillerton, and Erik LL Sonnhammer. "GRNbenchmark-a web server for benchmarking directed gene regulatory network inference methods." Nucleic Acids Research 50.W1 (2022): W398-W404.

• Seçilmiş, Deniz, et al. "Knowledge of the perturbation design is essential for accurate gene regulatory network inference." Scientific reports 12.1 (2022): 16531.

• Nakulugamuwa Gamage, Hasini, et al. "MICFuzzy: a maximal information content based fuzzy approach for reconstructing genetic networks." Plos one 18.7 (2023): e0288174.

Response:

Thank you for your comment. We extended the introduction with suggested references. Our manuscript now cites 70 different references, among which 16 are from 2020 or newer.

Comment #3:

In Section 3.1, please provide an example illustrating how these operators are performed?

Response:

Thank you for your suggestion. We included an example illustrating crossover and mutation operators in SAILoR. The included Fig 2 depicts topological modifications that arise from crossover and mutation, and corresponding adjacency matrices for the given example.

The provided figure caption explains genetic operators in detail:

"Two distinct child networks are produced by exchanging regulators of the second node. In the next step, each offspring network is mutated. Fig a) illustrates the topology of given networks. Fig b) illustrates corresponding adjacency matrices. Blue edges are modified by crossover. Edges marked with green and red are modified by mutation."

Comment #4:

I believe it would enhance reader comprehension if the description of the Jaccard index were exchanged with the description of the loss function in the manuscript. This adjustment would help readers better understand the concept.

Response:

We changed the order of the description of the Jaccard index with the definition of our loss function. We agree that this modification improves readability.

Comment #5:

In Figure 7, please provide more detailed descriptions of each bar, including their colors?

Response:

We improved the description of the figure to

"Performance improvements of SAILoR compared to dynGENIE3. Accuracy, Precision, Recall, F1 score, Bookmaker informedness (BM), and Matthew's correlation coefficient (MCC) improvements are calculated as a difference between the performance of SAILoR and the dynGENIE3 method. Blue boxes indicate improvements for networks with 16 nodes, orange boxes denote improvements for 32 node

networks, and green boxes represent 64 node network improvements. Results are obtained from 10 repetitions of 10 different networks for each network size. "

Comment #6:

The compared methods in the manuscript seem outdated. please compare SAILoR with some more recently proposed methods.

Response:

We additionally compared SAILoR with the more recent method LogBTF and accordingly extended Section 4.2

"The dynamic performance of LogBTF on our dataset is low, for which we hypothesize several reasons. First, Boolean threshold functions may not be as suitable for the representation of GRNs as disjunctive normal forms since Boolean threshold functions cannot describe an arbitrary logic function. For example, exclusive or, and logical equivalence cannot be represented with a Boolean threshold function. Second, LogBTF seems to be more sensitive to errors in our dataset, which arose from merging multiple time series data into a single time course. Errors often occur in real experimental data due to noisy conditions and measurement errors [62]. Finally, Li et al. [10] evaluated the dynamic performance of LogBTF on the training data, while 10-fold cross-validation was used in our case. This may additionally explain the discrepancy between our results and the results reported by Li et al."
